# Pupil-based arousal self-regulation: impact on physiological and affective responses to emotional stimuli

**DOI:** 10.1038/s41398-026-03937-3

**Published:** 2026-03-19

**Authors:** Jenny Imhof, Nora Maria Raschle, Nicole Wenderoth, Sarah Nadine Meissner

**Affiliations:** 1 Neural Control of Movement Laboratory, Department of Health Sciences and Technology, ETH Zurich, Gloriastrasse 37/39, 8092 Zurich, Switzerland; 2 Neuroscience Center Zurich, University and ETH Zurich, Winterthurerstrasse 190, 8057 Zurich, Switzerland; 3 Jacobs Center for Productive Youth Development, University of Zurich, Andreasstrasse 15, 8050 Zurich, Switzerland; 4 Future Health Technologies, Singapore-ETH Centre, Campus for Research Excellence and Technological Enterprise (CREATE), Singapore, 138602 Singapore; 5 Brain-Body Regulation Laboratory, Department of Health Sciences and Technology, ETH Zurich, Gloriastrasse 37/39, 8092 Zurich, Switzerland

**Keywords:** Human behaviour, Physiology

## Abstract

Pupil-based biofeedback has been shown to enable healthy participants to volitionally control locus coeruleus-mediated arousal. The locus coeruleus is considered a critical player in the central stress circuitry and dysfunctions in the system, causing dysregulated arousal levels, have been tightly linked to neuropsychiatric disorders such as anxiety and stress-related disorders and stress-induced cardiovascular vulnerability. Yet, it remains unclear whether physiological and self-rated affective responses to emotional stimuli are influenced by volitional control of arousal levels. In this study, healthy participants were presented with emotional (negative) or neutral sounds while they self-regulated (i.e., up- and downregulated) pupil size and pupil-linked arousal levels, a skill acquired through prior pupil-based biofeedback training. While no immediate online effect of such self-regulation on self-rated affect experience was observed, greater gains in pupil downregulation training predicted reduced affect experiences, particularly in response to negative sounds. Furthermore, larger pupil dilation responses to sounds were found during both pupil size up- and downregulation compared to a non-regulatory control condition, potentially indicating regulatory effort associated with pupil self-regulation. However, heart rate responses significantly decelerated during sound presentation and concurrent pupil size downregulation, suggesting parasympathetic dominance. These results provide the first evidence that pupil-based biofeedback training may modulate both self-rated and physiological responses to emotional sounds to a certain extent, highlighting its potential as a tool for reducing hyperarousal and hyperresponsivity to emotional sounds, as seen in anxiety and stress-related disorders, through pupil-linked arousal self-regulation.

## Introduction

The brain arousal levels greatly influence fundamental behaviors and mental well-being [1–5]. Multiple neuromodulatory systems are involved in arousal regulation with the locus coeruleus (LC) considered one of the primary modulators [6–8]. The LC projects to most cortical areas, orchestrating the distribution of noradrenaline (NA) to the central nervous system [6, 9], while receiving top-down input from the anterior cingulate cortex (ACC) and the orbitofrontal cortex (OFC) [10, 11]. Recent evidence implicates the LC-NA system as a key pathway causing dysregulated arousal states tightly linked to neuropsychiatric disorders including anxiety, depression and stress-related disorders [12–16]. Consistent with its role as a key hub in the central stress circuitry [5], the LC-NA system also mediates stress-induced cardiovascular vulnerability through projections to brainstem and spinal cord areas regulating autonomic functions [17, 18].

Modulating the human LC proves challenging due to its small size and location deep in the brainstem. Thus, measuring its activity and evaluating a successful modulation, remains challenging. Recently, we developed a pupil-based biofeedback (pupil-BF) approach that makes the brain’s arousal system accessible to volitional control [19]. This approach leverages the known co-dependence of (LC-driven) changes in arousal and pupil size under constant lighting conditions [1, 20–25]. Pupil-BF allows healthy individuals to volitionally increase [19, 26, 27] and decrease [19] (i.e., up- and downregulate) their pupil size while receiving real-time pupil diameter feedback. Importantly, using brainstem functional magnetic resonance imaging, we found that such self-regulation is associated with activity changes in the LC and, to a lesser extent, other arousal-regulating centers including dopaminergic and cholinergic regions [19]. Furthermore, markers of cortical excitability and cardiovascular arousal changed in tandem with pupil-based self-regulation [28].

During threatening situations, the LC releases NA, globally increasing arousal and reconfiguring large-scale brain networks [18, 24, 29–31]. Excessive or chronically increased LC activity is associated with maladaptive states, such as pathological anxiety [15, 32]. In rodents, stressors promote anxiety-like behaviors through LC-projections to the amygdala, where NA release alters function and consequently behavior [33]. Repeated exposure to such stimuli can increase anxiety-like behaviors [34] and LC sensitivity [35]. Direct evidence associating LC responsivity with psychopathology in humans remains limited. However, one study found that LC responses during an emotional conflict task *predict* an individual’s resilience to developing anxiety and depression symptoms under prolonged occupational stressors [36]. Furthermore, individuals diagnosed with post-traumatic stress disorder show stronger LC activity to sounds, which may mediate hyperarousal and hyperresponsiveness to sensory stimuli in these individuals [37]. Besides responses to the emotional content of stimuli (i.e., emotional reactivity [38–40]), there is first evidence that LC-NA activity indexed by pupil dilation, may be reflective of regulatory control [40], i.e., the modulation of response intensity, duration, or extent when facing an emotional situation (i.e., emotion regulation [41, 42]). Such regulatory control is crucial, and impairments have been considered a major risk factor for the development and maintenance of psychopathologies, including anxiety and stress-related disorders [43–45]. At the neural level, effortful emotion regulation has been associated with activity increases in cortical regions associated with response inhibition and cognitive control including the ventrolateral and dorsolateral prefrontal cortex, which may coincide with the downregulation of activity in emotion processing regions such as the amygdala or insula (for a review, see [46]). Interestingly, the LC-NA system is not only densely connected to these emotion processing regions [33, 47], but to cognitive control centers in the prefrontal cortex [10, 11], which may underly its potential role in regulatory control processes.

We have previously shown that participants can volitionally control LC-mediated arousal through pupil-BF. However, it remains unclear whether such volitional control affects self-rated affective and physiological responses induced by emotional stimuli. To address this, we combined pupil self-regulation, previously acquired through pupil-BF training, with concurrent presentation of emotional (i.e., negative) or neutral sounds. Participants were instructed to self-regulate pupil size prior to sound onset, allowing us to assess whether different pupil-based arousal states affect responses to emotional stimuli. First, we tested (i) whether such pupil self-regulation modulates self-reported affect experiences, especially following negative sounds, and (ii) whether pupil-BF training success predicts these experiences. Second, we examined whether physiological responses elicited by sound presentation were (iii) influenced by concurrent pupil self-regulation and (iv) linked to self-reported affect experiences.

## Methods

### Participants

We recruited 25 healthy participants (13 females; age 27±6 years) from a pool of previous participants [19, 28] or via online advertisement. Since this is, to our knowledge, the first study combining pupil-BF with the presentation of emotional stimuli, no statistical methods were used to pre-determine sample sizes. However, our sample size is similar or larger to those in previous studies investigating behavioral and physiological responses to emotion-inducing stimuli [38–40, 48]. All participants received standardized instructions and performed the measurements in a noise-shielded room allowing participants to focus on their task under controlled lighting conditions. Two participants did not complete all experimental sessions, resulting in a final sample size of *n* = 23 (12 females). All participants reported no neurological or psychiatric disorders, no intake of medication acting on the central nervous system and had normal or corrected-to-normal vision by contact lenses. On testing days, participants were asked to abstain from caffeine and the application of eye make-up. The study was conducted with the approval of the Cantonal Ethics Committee Zurich (KEK-ZH 2018-01078) and in accordance with the Declaration of Helsinki. Prior to participation, participants gave their written informed consent. Study participation was compensated (i.e., CHF 20/h).

### Pupil-BF (session 1-3)

Participants completed three pupil-BF training sessions as reported previously [19, 28]. Participants used mental strategies to up- or downregulate pupil size while receiving (real-time) pupil-BF (for further details, see Supplementary Material 1.1*)*.

### Emotion-inducing session (session 4)

After pupil-BF training, participants underwent a fourth session. If more than 10 days passed since the last pupil-BF session, participants received one re-training session identical to session 3 to ensure continued ability to modulate pupil size.

**Fig. 1:**
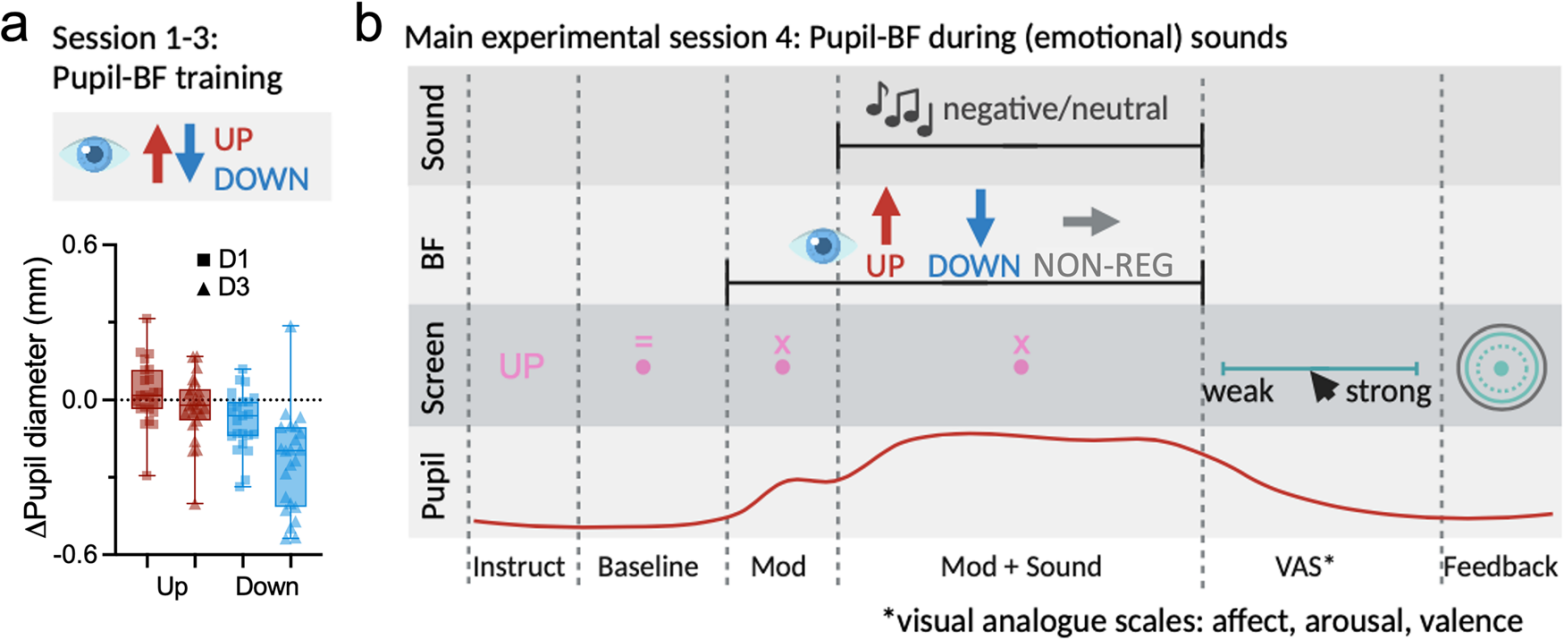
*Pupil-based biofeedback combined with emotional (i.e., negative) and neutral sound presentation.* **a** During pupil-based biofeedback participants apply mental strategies that have been shown to modulate arousal levels associated with the LC-NA system. During pupil-based biofeedback training sessions 1–3, participants (*n* = 23) were trained on three separate days to learn to upregulate and downregulate their own pupil size (30 UP/30 DOWN trials per session) indexing LC-mediated arousal. The lower panel shows pupil upregulation and downregulation performance, respectively, averaged across the 15 s modulation phase of session 1 and 3 (Pupil-BF training). We found a significant improvement from session 1 to session 3 for downregulation (paired samples t-test: t(22) = 4.91; *p* < 0.001) but not for upregulation of pupil size (*p* = 0.14). **b** Exemplary trial of the emotion session (session 4). Participants regulated pupil or executed a non-regulatory control task while listening to emotion-inducing, negative versus neutral sounds. Each trial started with a 3 s baseline phase, followed by 2 s of modulation (UP, DOWN, NON-REG) with no sounds, followed by 6 s of modulation (UP, DOWN, NON-REG) while negative or neutral sounds were played. An unrestricted rating phase followed during which participants self-reported on experienced affect intensity, arousal and valence levels using a visual analogue scale. Finally, 1.5 s of color-coded post-trial performance feedback was displayed reflecting the pupil self-regulation success (in green if successful and in magenta if unsuccessful). During non-regulatory trials, performance feedback was replaced by a pink fixation dot to keep trial timings similar between conditions. Throughout the trials, heart rate, respiration, and pupil size were continuously recorded. BF = biofeedback; VAS = visual analogue scale. Instruct = Instruction. Boxplots indicate median (centre line), 25th and 75th percentiles (box), and maximum and minimum values (whiskers). Squares and triangles represent individual participants. Tests were two-tailed and corrected for multiple comparisons.

During the fourth session, participants applied pupil self-regulation strategies (i.e., UP and DOWN) or performed a non-regulatory control (NON-REG) task while listening to 60 negative and 60 neutral sounds from the IADS-2 [49] databank (Fig. 1b). Each condition was presented in blocks of 5 trials. These blocks were presented in pseudorandomized manner. For a detailed description of the sounds and block structure, see Supplementary Material *1.2**Emotion-inducing task: Stimuli & block design*. Each trial comprised of an instruction, a pupil baseline measurement, a pre-sound modulation phase, a modulation phase during sound presentation, self-ratings, and a break (illustrated in Fig. 1b). During the baseline phase, participants counted backwards from 100 in steps of four to ensure a controlled mental state and to avoid early application of modulation strategies. Participants were then either self-regulating their pupil size (applying strategies acquired during pupil-BF training to induce different LC-mediated arousal states prior to sound onset) or to continue counting backwards (non-regulatory control). This aimed to investigate whether varying pupil-based arousal states result in distinct physiological and subjective responses to emotionally negative stimuli. Then, a negative or neutral sound was played, during which participants continued to up- or downregulate pupil size or listened (NON-REG). Following sound presentation, participants intuitively rated the intensity of their experienced affect (weak-strong) evoked by the sound, arousal (calm-arousing) and valence (unpleasant-pleasant) on a continuous visual analogue scale (VAS) using the right and left keyboard arrow bars. Then, color-coded post-trial feedback appeared: Successful modulation (i.e., larger mean pupil size for UP/smaller for DOWN compared to baseline) was indicated by a green circle, indicating the average change in pupil size relative to the measured baseline shown as dashed circle. Unsuccessful modulation was signaled by a magenta circle. The feedback was calculated as described for training session 3 (Supplementary Material 1.1.). Between blocks of 5 trials, participants could take self-paced breaks. Colors on the screen were isoluminant to the grey background and the same as used during the third day of pupil-BF training (Supplementary Material 1.1.).

### Pupil and cardiovascular measurements

Pupil size, electrocardiography (ECG) and respiratory data was recorded throughout all sessions. Respiratory data are not reported here. For details see Supplementary Material *1.3**Pupil size and cardiovascular measurements*.

### Questionnaires

To assess participants’ emotion regulation strategies, the emotion regulation questionnaire (ERQ) targeting the habitual use of expressive suppression and cognitive reappraisal was used [50, 51]. State and trait anxiety were assessed using the state and trait anxiety inventory (STAI) [52]. While the ERQ and the trait STAI were assessed during pupil-BF training or re-training, state anxiety was assessed on the day of the emotion-inducing session prior to the experimental task (see Supplementary Material 1.8 and 1.9, and Supplementary Fig. 7).

### Self-report data analysis

As a primary outcome, we investigated the intensity of affect experience for all self-regulation and non-regulatory control conditions (arousal and valence ratings are secondary outcomes and reported in Supplementary Fig. 1). All VAS scores (0–100) were extracted and averaged across negative and neutral sound trials for each condition (UP, DOWN, NON-REG) and each participant.

If not reported otherwise throughout the manuscript, statistical analyses were performed using IBM SPSS 28.0.1.1 (IBM Corporation, Armonk, NY, USA) and all analyses were corrected for multiple comparisons using sequential Bonferroni correction [53]. To investigate the effect of self-regulation and sound condition on self-reported affect experiences, we conducted a repeated measures ANOVA with the within-subjects factors self-regulation (UP versus DOWN versus NON-REG) and sound (negative versus neutral). Sphericity was assessed using Mauchly’s sphericity test and violations were accounted for with the Greenhouse-Geisser correction. Since some residuals of the arousal and valence ratings deviated significantly from normal distribution (Shapiro Wilk: *p* < 0.05), we conducted non-parametric tests for these outcomes. First, we compared neutral versus negative sound ratings for each self-regulation condition using Wilcoxon signed rank tests. Then, we conducted Friedman ANOVAs for arousal and valence ratings, respectively, where we tested the effect of self-regulation in each sound condition separately. To test for potential interaction effects, a Friedman ANOVA was conducted on the difference between negative and neutral sounds with the within-subjects factor self-regulation condition (UP versus DOWN versus NON-REG). In case of significant effects, we derived post-hoc *p-*values.

### Pupil data offline processing and analysis

Pupil data was pre-processed as described in Meissner et al. [19] (see Supplementary Methods *1.4**Offline processing of pupil data* for details).

#### Pupil-BF training data

For pupil-BF data, we averaged the baseline-corrected pupil diameter time series during modulation phases across the UP and DOWN condition at the beginning (session 1) and end of training (session 3) for each participant.

To investigate whether participants significantly improved their pupil self-regulation ability across the course of training, we conducted a repeated measures ANOVA with the within-subjects factors session (1 versus 3) and self-regulation (UP versus DOWN). In case of a significant interaction, we derived post-hoc *p-*values. Pupil data of session 3 was reported previously in the context of cortical arousal markers during pupil-BF [28].

#### Pupil size changes during emotion-induction: Pupil self-regulation and pupil dilation responses to sounds

Baseline-corrected pupil size time series were computed for the final second of the baseline phase, pre-sound modulation and during sound presentation. Data was averaged for each sound (neutral and negative) and self-regulation condition (UP, DOWN and NON-REG), leading to six different time series. Then, mean pupil size of the last 200 ms before sound onset (averaged across sound conditions) was analyzed via a Friedman ANOVA to assess whether participants successfully modulated pupil size prior to sounds.

To examine whether pupil dilation responses varied with self-regulation and sound condition, pupil time series were baseline-corrected to the last 200 ms before sound onset and analyzed using the MATLAB-based SPM1D toolbox for one dimensional data (SPM1D version M.0.4.11; https://spm1d.org/ [19, 54, 55]). Since some residuals violated normality (D’Agostino-Pearson K² test; *p* < 0.05), the data was subjected to a non-parametric permutation-based two-way repeated-measures ANOVA with the within-subjects factors sound (negative vs. neutral) and self-regulation (UP vs. DOWN vs. NON-REG). SPM1D post-hoc comparisons were performed using non-parametric permutation-based two-sided paired t-tests with Bonferroni correction.

To explore differences in pupil dilation velocity depending on self-regulation and sound condition, we computed the first derivative of baseline-corrected pupil time series [46] (for details, see Supplementary Methods *1.5Pupil time series analysis*). Derivative-based time series were averaged per participant within each self-regulation and sound condition. Statistical differences were assessed using the same SPM1D approach as described in the previous paragraph. As a secondary analysis, we investigated whether pupil-BF training success was linked to derivative-extracted measures. For each sound and self-regulation condition, peak pupil dilation velocity and latency to peak velocity were extracted from the first derivative of the baseline-corrected pupil time series to sounds. To reduce the number of statistical comparisons, pupil-BF training success was based on the difference in pupil modulation indices (MI, the time series during downregulation subtracted from the time series during upregulation (UP-DOWN), averaged across the 15 s modulation phase) between session 1 and 3 in this secondary analysis. This modulation index was correlated with the extracted peak and latency variables for each sound and self-regulation condition. Since most of the residuals, especially those of the latency variables, were violating normality, we computed Spearman’s Rho correlation coefficients.

### Cardiac data (pre-)processing and analyses

Cardiac data pre-processing followed Meissner et al. [19] and is described in Supplementary Methods *1.6**. Cardiac data (pre-)processing*. Relative heart rate changes were calculated for each time point:

$$\% -{heart}\,{rate}\,{change}=100* \frac{{heart}\,{rate}-{mean}\,{heart}\,{rate}\,{Baseline}}{{mean}\,{heart}\,{rate}\,{Baseline}}$$

Then, we calculated the average change in heart rate for each sound and self-regulation condition during sound presentation as compared to the pre-sound modulation phase.

To investigate whether pupil self-regulation systematically modulated heart rate in response to negative and neutral sounds, we subjected average heart rate changes to sounds for each self-regulation (UP versus DOWN versus NON-REG) and sound condition (neutral versus negative) to a repeated-measures ANOVA. Sphericity was assessed using Mauchly’s sphericity test and violations were accounted for with the Greenhouse-Geisser correction. In case of significant effects, we derived post-hoc *p*-values. For additional control analyses of the baseline phase, refer to Supplementary Methods *1.7**. Control analysis of the average baseline values for heart rate and pupil size* [53].

### Self-regulation success and subjective affect experiences

To further investigate our research question of whether pupil-BF training success is related to self-rated affect experiences evoked by sounds, and especially negative sounds, we conducted three stepwise regression analyses. Affect ratings averaged within the respective self-regulation conditions (UP, DOWN, and NON-REG) for negative sounds were entered as dependent variables. To investigate differential effects of up- and downregulation training on self-ratings, improvements in pupil size upregulation (i.e., UP_session3_-UP_session1_) and downregulation (i.e., DOWN_session3_-DOWN_session1_) were entered as predictors, while controlling for participants’ sex [53]. Three additional exploratory stepwise regression analyses were calculated for the ratings of *neutral* sounds using the same predictors.

Even though self-reported emotion measures are the outcome of choice in behavioural emotion regulation studies and are considered stable and reliable [46], we implemented in an additional analysis an attenuation correction based on the modified Spearman’s attenuation equation [56] to account for possibly limited retest-reliability in our sample (maximum assumed reliability = 0.9; see Supplementary Methods *1.10**Spearman’s attenuation correction*).

### Pupil dilation responses and subjective affect experiences

Finally, we tested the hypothesis that maximum pupil dilation responses to sounds extracted from the baseline-corrected pupil time series are predictive of self-reported affect experiences induced by negative sounds. Here, we conducted two separate stepwise regression analyses with the differences in affect experiences (i.e., UP – NON-REG and NON-REG – DOWN) as dependent variable and pupil dilation differences (i.e., UP – NON-REG and NON-REG – DOWN) as predictors while controlling for sex. Exploratory control analyses were repeated for neutral sound conditions implementing the same predictors and dependent variables. In case variables were not normally distributed, Spearman’s Rho correlation coefficients were calculated.

Additionally, we aimed to investigate whether pupil dilation velocity-related measures extracted from the first derivative of baseline-corrected pupil time series were significantly related to affect experiences. However, since peak velocity-derived measures consistently correlated positively with maximum pupil dilation responses (all correlation coefficients > 0.52), we refrained from additional analyses since the unique contributions of the different variables could not necessarily be disentangled (e.g., multicollinearity issue).

## Results

### Pupil-BF training effects

To determine whether participants were able to improve their self-regulation skills during training, we conducted a repeated-measures ANOVA with the factor self-regulation (UP versus DOWN) and session (D1 versus D3). This analysis revealed significant main effects of session and self-regulation, which can best be interpreted in light of a significant interaction between self-regulation and session (*F*(1,22) = 9.77; *p* = 0.005; η_p_^2^ = 0.31; 95%-confidence interval (CI)η_p_^2^ [0.03;0.54]; Fig. 1a). This interaction was driven by significant improvements in downregulation (*t*(22) = 4.91; *p* < 0.001; *d* = 1.02; 95%-CId = [0.51;1.52]) but not upregulation (*p* = 0.14) across training sessions (for pupil modulation indices of the same training dataset, see [28]).

These findings indicate that participants were able to improve their self-regulation skills across pupil-BF training. Here, this was mainly driven by improved *downregulation*.

### Pupil self-regulation and affect experiences

#### Online pupil self-regulation does not influence affect experiences

**Fig. 2:**
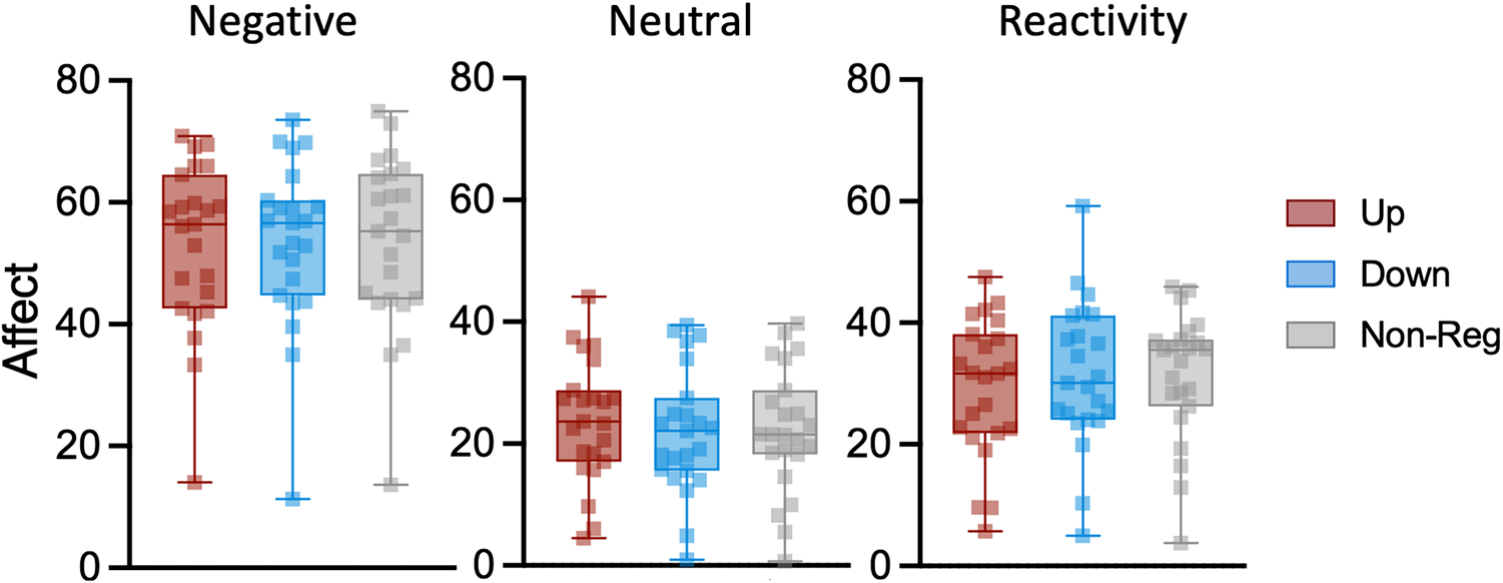
*Behavioral self-ratings.* Intensity of affect experience indicated on a visual analogue scale following negative (left panel) and neutral sound presentation (middle panel) during upregulation (red), downregulation (blue), and non-regulatory control trials (grey). Whereas we found a significant effect of sound (i.e., stronger affect experiences for negative as compared to neutral sounds), we did not find a significant effect of self-regulation condition or interaction effect. The right panel indicates the difference in self-ratings between negative and neutral sound conditions, thus reactivity. Boxplots indicate median (centre line), 25th and 75th percentiles (box), and maximum and minimum values (whiskers). Squares indicate individual participants.

A repeated-measures ANOVA conducted to test our primary hypothesis that affect experiences following negative sounds are influenced by pupil-based arousal self-regulation revealed a main effect of sound (*F*(1,22) = 206.47; *p* < 0.001; η_p_^2^ = 0.90; 95%-CIη_p_^2^ [0.80;0.94]) with significantly higher ratings for negative as compared to neutral sounds (Fig. 2). In contrast to our assumptions, no other main effect or interaction reached significance (all *p* > 0.35), indicating that online pupil self-regulation had no significant influence on affect experiences following neutral or negative sounds at group level. Similar results were obtained when adding sex as a covariate to the model (main effect sound: (*F*(1,21) = 11.62; *p* = 0.003; η_p_^2^ = 0.36; 95%-CIη_p_^2^ [0.06;0.58]).

Whilst our findings validated that negative sounds were more arousing and unpleasant (Supplementary Fig. 1) and elicited stronger affect experiences (Fig. 2), we did not find consistent effects of online pupil self-regulation on subjective affect experiences on a group level.

#### Pupil self-regulation training success predicts affect experiences induced by negative sounds

**Fig. 3:**
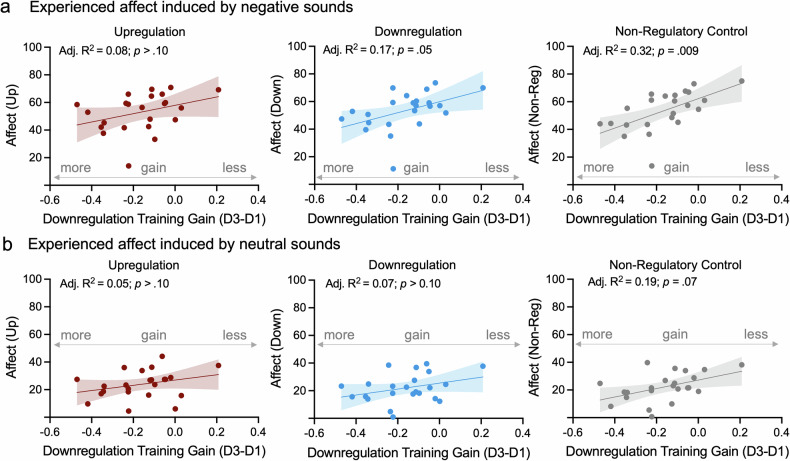
*Self-regulation training is associated with experienced affect, especially when negative sounds are presented.* **a** Linear regression analyses revealed that improvement of pupil size downregulation (i.e., the more negative the better) from session 1 to session 3 but not pupil size upregulation significantly predicted the intensity of affect experiences evoked by negative sounds during downregulation (middle panel) and non-regulatory control trials (right panel) but not during upregulation (left panel). **b** Additional exploratory analyses on neutral sounds revealed a similar trend-level effect of downregulation (but not upregulation) training gain only in non-regulatory control trials but not during down- or upregulation. Shaded areas indicate the 95%-Confidence Interval. All *p*-values were corrected for multiple comparisons using sequential Bonferroni correction.

The lack of significant effects of concurrent pupil self-regulation on affect experiences may be due to the variability in these ratings and self-regulation success. We therefore tested the hypothesis that pupil-BF training success predicts interindividual differences in these ratings. Stepwise regression analyses revealed that downregulation training gain (i.e., session3-session1; more improvement reflected in more negative values) was predictive of *reduced* subjective affect ratings towards negative sounds during downregulation (R = 0.46; adjusted R^2^ = 0.17; *p* = 0.05; Fig. 3a, middle panel) and non-regulatory control trials (R = 0.59; adjusted R^2^ = 0.32; *p* = 0.009; Fig. 3a, right panel), however, not during upregulation trials (*p* ≥ 0.35; Fig. 3a, left panel). Upregulation gain did not significantly add to the explained variance of self-ratings (all *p* ≥ 0.68). To account for possible limitations in test-retest reliability of the variables, we used the modified Spearman’s attenuation equation [56] leading to a maximum R^2^_adj_ = 0.26 for negative sounds during downregulation and a maximum R^2^_adj_ = 0.50 for control trials in the current cohort.

Exploratory analyses on neutral sounds revealed that self-regulation training gain did not significantly predict subjective affect experiences during up- or downregulation (all *p* ≥ 0.10; Fig. 3b left and middle panel). For non-regulatory control trials, we found a trend-level effect where better downregulation training gain predicted *reduced* subjective affect experiences (adjusted R^2^ = 0.19; *p* = 0.07; Fig. 3b, right panel).

Taken together, larger *downregulation* training improvements were related to reduced intensity of affect experiences, especially when induced by negative sounds.

### Pupil self-regulation influences pupil dilation responses independently of sound condition

**Fig. 4:**
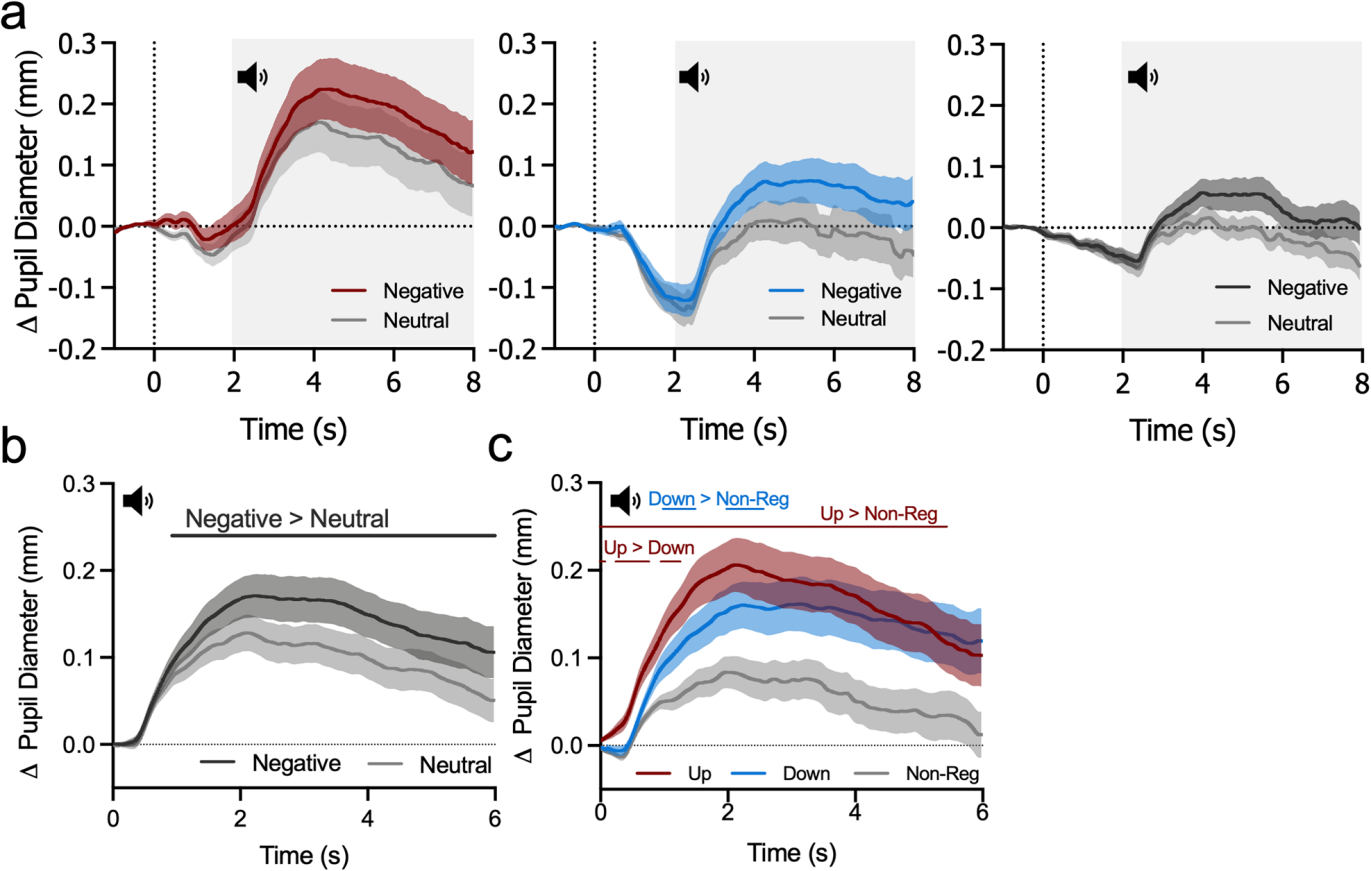
*Pupil self-regulation and dilation responses to sounds.* **a** Average changes in pupil size (i.e., baseline-corrected) to negative and neutral sounds during upregulation (left panel), downregulation (middle panel) and non-regulatory control trials (right panel) are shown. Whereas self-regulation starts at t = 0 s (dashed vertical line), sound presentation starts at t = 2 s (indicated by the sound icon and grey box). **b** Time series of pupil dilation responses evoked by negative (dark grey) and neutral (light grey) sounds averaged across all self-regulation conditions. Significantly larger dilation response for negative as compared to neutral sounds (SPM1D repeated-measures ANOVA main effect of sound category: F* = 8.71; one sig. cluster, *p* < 0.001). **c** Time series of pupil dilation responses to sounds during self-regulation (upregulation in red, downregulation in blue, and non-regulatory control trials in grey). Upregulation trials evoked significantly larger pupil dilation than non-regulatory control trials (t* = 3.07; one cluster, *p* = 0.001; Bonferroni-corrected α = 0.0167). Additionally, upregulation evoked greater pupil dilation than downregulation (t* = 2.98; *p* = 0.002), and downregulation evoked greater pupil dilation than non-regulatory control (t* = 3.167; *p* = 0.001). Shaded areas indicate s.e.m. Black and colored horizontal lines in panel (b) and (c) denote time clusters with significant differences between self-regulation conditions.

We observed that both negative and neutral sounds elicit pupil dilation responses (Fig. 4a). The SPM1D repeated-measures ANOVA of pupil dilation responses (baseline-corrected to the last 200 ms before sound onset) revealed a significant main effect of sound, with significantly larger pupil dilation responses to negative as compared to neutral sounds between 0.9–6 s (F* = 8.712; one significant cluster, *p* = 0.001; Fig. 4b and Supplementary Fig. 3a). Furthermore, a significant main effect of self-regulation was detected between 0–5.4 s and 5.9-6 (F* = 5.51; two significant clusters with *p* = 0.001; Fig. 4c and Supplementary Fig. 3a). Importantly, no significant interaction was observed (*p* > 0.05; Supplementary Fig. 3a), suggesting that the effect of self-regulation on pupil dilation did not depend on the sound condition. Therefore, post-hoc comparisons were conducted collapsed across sound conditions, revealing that pupil dilation responses were significantly larger during upregulation compared to non-regulatory control trials from 0–5.4 s (t* = 3.07, one sig. cluster with *p* = 0.001; Bonferroni-corrected α = 0.0167: Fig. 4c). Additionally, three clusters between 0–1.3 s showed significantly larger pupil dilation during upregulation than downregulation (t* = 2.98, all clusters *p* = 0.002), and two clusters between 1–2.6 s indicated larger pupil dilation during downregulation compared to non-regulatory control trials (t* = 3.16, both clusters *p* = 0.001). For detailed outputs of the SPM1D ANOVA including post-hoc tests, see Supplementary Fig. 3.

Next, we tested whether pupil dilation velocity differs during sound presentation depending on self-regulation and sound condition. The analysis revealed a significant main effect of self-regulation (F* = 8.39; two significant clusters with *p* = 0.001; Supplementary Fig. 4) with significantly faster pupil dilation changes during upregulation than non-regulatory control trials for two brief clusters close to sound onset (t* = 4.22; two clusters 0–0.008 s and 0.5–0.6 s with *p* = 0.008 and *p* = 0.006 respectively; Bonferroni-corrected α = 0.0167). No other significant effects were found (all *p* > 0.05; Supplementary Fig. 4). Our secondary analysis further revealed that peak pupil dilation velocity to negative sounds during non-regulatory control trials was positively related to pupil-BF training gain (i.e., MI_session3_-MI_session1_; Rho = 0.55, *p* = 0.036), indicating that the larger the training gain, the faster the pupil dilation to sounds. For self-regulation trials, these variables were largely unrelated (all *p* > 0.15 corrected; Supplementary Fig. 5ab). However, this was mainly driven by one outlier with a very low training success. Removing this outlier, we found significant positive relationships between pupil-BF training gain and peak pupil velocity for all self-regulation and sound conditions (UP_neg_: Rho = 0.43; *p* = 0.045; UP_neu_: Rho = 0.65; *p* < 0.001; DOWN_neg_: Rho = 0.51; *p* = 0.03*;* DOWN_neu_: Rho = 0.54; *p* = 0.03; NON-REG_neg_: Rho = 0.78; *p* < 0.001; NON-REG_neu_: Rho = 0.64; *p* = 0.004; Supplementary Fig. 5cd; there were no significant relationships with latency to peak velocities; all *p* > 0.08 uncorrected).

As participants began to self-regulate pupil size prior to sound onset, we additionally examined whether pupil size baseline-corrected to pre-modulation differed between upregulation, downregulation, and non-regulatory trials prior to sound onset (i.e., averaged across 200 ms before sound onset). Analyses revealed a significant main effect of self-regulation (χ^2^ = 12.09; *p* = 0.002), mainly driven by a stronger decrease during downregulation as compared to upregulation and (z = −3.13; *p* = 0.006; *r* = *−*0.65) non-regulatory control trials (z = −2.89; *p* = 0.008; *r* = *−*0.60). Upregulation was not significantly different to non-regulatory control trials 200 ms before tone onset (z = 1.64; *p* = 0.10; *r* = 0.34; Supplementary Fig. 6).

In summary, our findings indicate that, despite the significant effect of self-regulation on pupil dilation responses to sounds, this response modulation was not specific to emotional sounds.

### Pupil self-regulation modulates heart rate responses to sounds

**Fig. 5:**
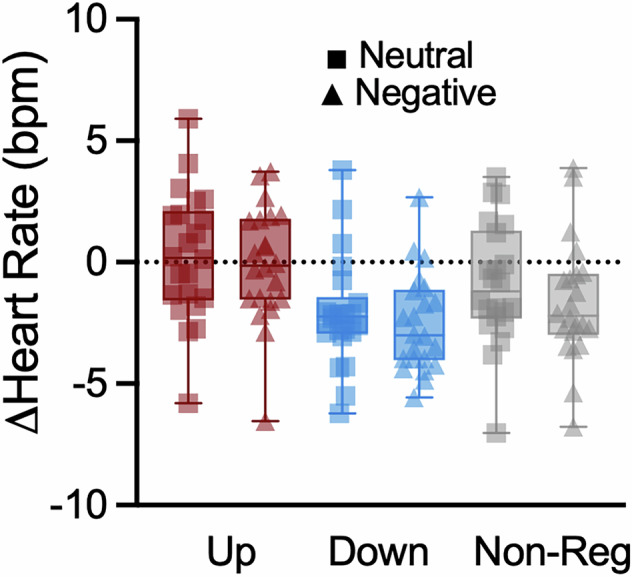
*Effects of pupil self-regulation on heart rate responses.* Changes in heart rate evoked by negative and neutral sounds during upregulation (red), downregulation (blue) and non-regulatory control trials (grey) across all participants (*n* = 22). Heart rate deceleration significantly increased from upregulation to non-regulatory control to downregulation trials, independent of sound category. Boxplots indicate median (centre), 25^th^ and 75^th^ percentiles (box), maximum and minimum values (whiskers). Squares and triangles represent individual data.

Previously, we found that pupil self-regulation is associated with concurrent heart rate changes: pupil size upregulation led to an increase in heart rate as compared to downregulation [19, 28]. Here, we tested whether pupil self-regulation leads to a modulation of heart rate during sound presentation. We found a significant main effect of pupil self-regulation on heart rate responses (*F*(2,42) = 14.53; *p* < 0.001; η_p_^2^ = 0.41; 95%-CIη_p_^2^ [0.16;0.56]; Fig. 5; adding sex as a covariate, a similar effect was observed; *F*(2,40) = 3.76; *p* = 0.03; η_p_^2^ = 0.16; 95%-CIη_p_^2^ [0;0.33]). This was mainly driven by a significant *decrease* in heart rate during downregulation as compared to non-regulatory control (*t*(21) = −2.67; *p* = 0.014; *d* = −0.57; 95%-CId [−1.02;−0.11]) and upregulation trials (*t*(21) = 5.55; *p* < 0.001; *d* = 1.18; 95%-CId [0.63;1.72]; difference UP vs. NON-REG: *t*(21) = 2.67; *p* = 0.028; *d* = 0.57; 95%-CId [0.11;1.02]). Surprisingly, there was no significant effect of sound condition on heart rate (*p* = 0.09).

### Pupil dilation responses and affect experiences

**Fig. 6:**
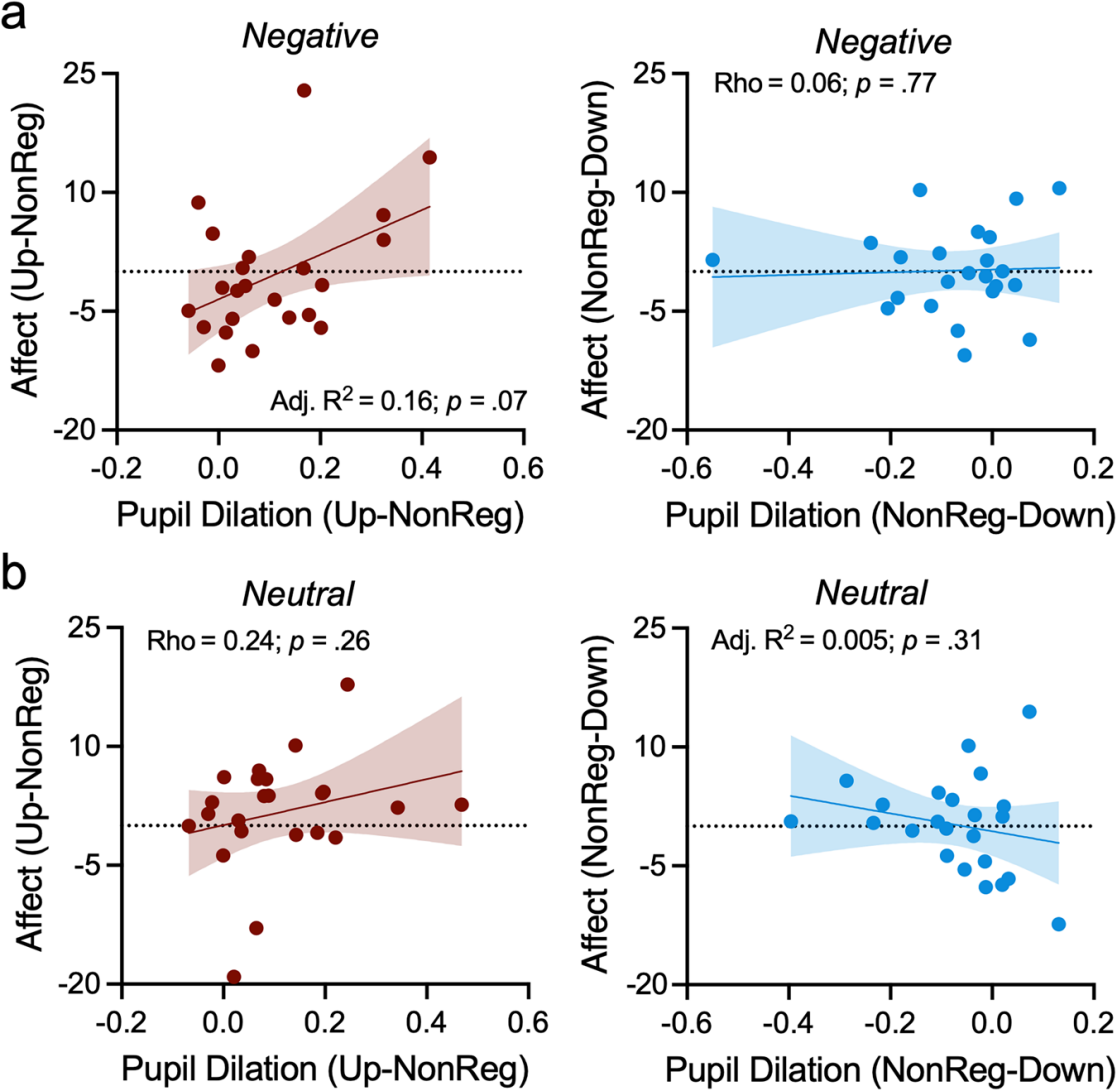
*Pupil dilation responses and experienced affect.* Maximum pupil dilation responses during upregulation (as compared to non-regulatory control trials) was linked to (i.e., trend-level effect after correcting for multiple comparisons) subjective experiences of affect induced by negative (**a**, left panel) but not neutral sounds (**b**, left panel) during upregulation. There was no significant link between pupil dilation responses during downregulation (as compared to non-regulatory control trials) and subjective experiences of affect, neither for negative (**a**, right panel) nor for neutral sounds (**b**, right panel). Shaded areas indicate the 95%-CI.

Finally, we investigated whether pupil dilation responses to sounds were related to affect experiences. Linear regression analyses did not reveal significant effects. There was only a trend-level prediction of stronger affect experiences to negative sounds during upregulation as compared to non-regulatory control trials by pupil dilation responses after correcting for multiple comparisons (R = 0.44; adjusted R^2^ = 0.16; *p* = 0.07; Fig. 6a left panel).

No other analyses (i.e., Fig 6ab) revealed a significant association between physiological responses and affect experiences (all *p* ≥ 0.26).

## Discussion

We previously demonstrated that participants can learn to volitionally control LC-mediated arousal through pupil-BF [19]. Here, we investigated whether engaging the arousal system through self-regulation affects responses to negative stimuli, previously linked to LC-NA system activity. Our three main results provide partial support for a link between arousal self-regulation and self-rated and physiological responses to sounds: First, even though concurrent pupil self-regulation did not influence affect experiences, pupil-BF training success predicted affect experiences induced by negative sounds (Fig. 3). Second, pupil self-regulation influenced pupil dilation responses to sounds. While these responses were larger for negative as compared to neutral sounds, they were further enhanced by pupil self-regulation as compared to non-regulatory control trials (Fig. 4) and may reflect regulatory effort during sound presentation. Interestingly, heart rate responses to sounds deviated from pupil dilation responses such that we found increased heart rate *deceleration* during downregulation as compared to non-regulatory control and upregulation (Fig. 5). This may suggest stronger parasympathetic dominance during pupil downregulation. Third, pupil dilation responses to sounds were only partially related to affect experiences induced by these sounds (Fig. 6).

In the present study, we observed a significant pupil-BF training effect (i.e., larger pupil self-regulation in session 3 than session 1), mainly driven by improved downregulation. The absence of an upregulation training effect was unexpected but may reflect the longer self-regulation period in session 3 (30 s vs 15 s) due to additional blood pressure measurements not reported here. Although only the first 15 s of each modulation phase were analysed, prolonged modulation times can influence pupil size upregulation, also affecting earlier time windows [57].

Our primary hypothesis that pupil self-regulation modulates concurrent affect experiences induced by negative sounds was not confirmed: Affect experiences did not significantly differ between self-regulation and non-regulatory control conditions (Fig. 2). This contrasts classical *behavioral* emotion regulation paradigms, where such ratings are usually lower during explicit behavioral downregulation (e.g., cognitive reappraisal) than during upregulation or control [40, 48, 58]. However, pupil downregulation training success *predicted* individuals’ affect experiences induced by negative sounds. Participants who showed larger downregulation improvements were less affected by negative sounds when they downregulated pupil size (Fig. 3). In general, the interpretation of these results warrants caution to the relatively limited sample size and needs replication in larger studies. One speculation is that the ability to downregulate pupil-linked arousal influences how negative sounds are perceived and showcases individual differences in acquisition ease and benefit of pupil-BF training. Interestingly, pupil-downregulation training success not only predicted negative affect experiences during downregulation but also during non-regulatory control trials. Whether this link indicates an inherent self-regulation ability which is reflected in physiological (i.e., pupil-BF training success) and affective responses, or rather implicit activation of acquired strategies during control trials remains to be determined. In contrast, upregulation training success was not related to (e.g., enhanced) affect experiences. The interpretation is limited by the lack of significant improvements in upregulation across pupil-BF training. Nevertheless, the overall findings, specifically the link between successful downregulation of (pupil-based) arousal and reduced intensity of affect experiences following negative stimuli, are promising. As irregular responses to emotional situations are a major risk factor for the development and maintenance of anxiety- and stress-related disorders [43–45], future work should test whether training-induced downregulation of these responses could help mitigate such conditions.

Looking at pupil dilation corrected to pre-modulation baseline phases (Fig. 4a), pupil size was generally larger during upregulation as compared to downregulation and non-regulatory control trials, suggesting higher pupil-linked arousal during upregulation. Furthermore, even though participants successfully downregulated pupil size pre-sound onset (Supplementary Fig. 6), pupil-linked arousal reached a relatively similar level as during non-regulatory control trials (Fig. 4a). Statistical comparisons of pupil dilation responses *after* sound onset revealed larger responses during the presentation of negative than neutral sounds (Fig. 4b), replicating previous findings of pupil dilation responses reflecting emotional reactivity [38–40]. These responses were further influenced by pupil self-regulation, independent of neutral or negative sounds: pupil dilation was larger during pupil size up- and downregulation as compared to non-regulatory control trials (Fig. 4c). Especially for upregulation trials, significant differences were present over almost the entire sound presentation. Enhanced pupil dilation responses during self-regulation compared to non-regulatory control trials are in line with a study by Maier and Grueschow [40], reporting larger pupil responses to emotional stimuli during the application of *behavioral* emotion regulation strategies as compared to pure perception trials. It may thus be hypothesized that regulatory effort involves the pupil-linked arousal system leading to increased dilation responses [40]. In our data, this proposed regulatory effort effect was reflected early in the pupil dilation response (Supplementary Fig. 3a), which may not be surprising given that participants started self-regulation 2 s before sound onset. In contrast, the effect of sound reached significance around 0.9 s after sound onset and evolved over time. Whether this may differ if sound and self-regulation onset would be temporally aligned, as for behavioral emotion regulation paradigms, can only speculated on. To get a deeper understanding of the contribution of different central autonomic pathways to such pupil responses, a previous study used pharmacological manipulation at the pupillary muscles and different lighting conditions during sustained processing. Interestingly, they found evidence that especially parasympathetic inhibition may mediate effort-linked pupil dilation responses [59]. This is consistent with earlier work highlighting the role of parasympathetic pathways in pupil responses to sensory stimulation [60].

While pupil dilation responses were significantly affected by self-regulation and sound condition, pupil dilation velocity was only influenced by self-regulation with significantly faster dilation during upregulation compared to non-regulatory control trials (Supplementary Fig. 4). In a secondary analysis, we investigated whether interindividual differences in pupil-BF training gain may be linked to differences in pupil velocity, i.e., whether participants learning to self-regulate better show faster dilation responses. We found pupil-BF training success indeed to be linked to peak dilation velocity independent of sound and self-regulation condition (Supplementary Fig. 5). It remains to be determined whether such a link indicates a general physiological flexibility which is reflected both in pupil-BF training success and pupil responses to sensory signals or rather indicates that acquired internal self-regulation changes such responses to external stimuli.

Consistent with the role of the LC-NA system in controlling autonomic activity through projections to cardiovascular regulatory structures [61], pupil self-regulation influenced heart rate responses to sounds (Fig. 5). Previously, heart rate was significantly reduced during pupil size down- compared to upregulation [19, 28]. Here, we found stronger heart rate deceleration during downregulation as compared to upregulation and non-regulatory control trials, even when participants simultaneously attended to arousing, unpleasant sounds. This finding may be indicative of a parasympathetic dominance during downregulation, making it tempting to speculate that such downregulation may buffer cardiovascular responses to aversive stimuli. This is especially interesting in the light of increased pupil dilation responses to sounds during pupil self-regulation that likely reflect regulatory effort. A previous study comparing meditators to non-meditators found reduced heart rates in meditators during neutral and negative stimuli presentation [62], which is consistent with the current results and the notion that parasympathetic activity increases during meditation practice [63]. A similar heart rate deceleration has been further linked to enhanced performance in response to threats possibly mediated by increased attention and response preparation processes [64–67], indicating that the function of deceleration may lie in actively facilitating accurate decision making to optimize coping under threat [65].

In contrast to a previous study linking larger pupil dilation responses to lower affect experiences during *behavioral* downregulation [40], we only found a trend-level effect (after correcting for multiple comparisons) of pupil size changes during upregulation compared to non-regulatory control trials to be predictive of *increased* affect experiences induced by negative sounds (Fig. 6a). This may suggest that deliberate upregulation of the brain’s arousal system leads to stronger affect experiences of negative situations. Such an increase may be influenced by negative emotional imagery used by some participants to upregulate pupil size. Pupil dilation responses during downregulation compared to non-regulatory control trials were not significantly related to affect experiences induced by negative sounds. The difference to previous results [40] might be driven by the fact that, here, participants were deliberately self-regulating pupil size as compared to the naturally occurring pupil responses linked to the explicit use of cognitive control strategies during affective sound presentation [40].

There are several study limitations. First, participants were instructed to self-regulate pupil size *prior* to sound onset, thus, the emotional state was induced when participants were already applying self-regulation. This differs from classical emotion regulation studies [40, 58, 68] and was implemented to be able to test whether different pupil-based arousal states when encountering emotional stimuli would lead to different responses. However, future studies inducing emotional states followed by self-regulation could help elucidating the utility of pupil self-regulation to influence responses to negative stimuli. Finally, even though we have previously shown that pupil self-regulation targets LC activity, we did not investigate LC activity directly here. To draw direct conclusions on the interaction between emotion regulation systems with the LC-NA system, future neuroimaging studies are required.

In summary, our study demonstrates that pupil-BF, linked to activity changes in arousal-regulating centers and particularly the LC, modulates subjective and physiological responses to negative and neutral sounds. While greater pupil downregulation training improvements predicted reduced affect experiences, especially for negative sounds, both pupil up- and downregulation increased pupil dilations responses to sounds, potentially reflecting increased regulatory effort. However, heart rate significantly decelarated during downregulation, which may be indicative of parasympathetic dominance. Although this study provides an initial proof-of-concept, future research is needed to explore the clinical potential of pupil-BF. Of specific relevance is its effectiveness in modulating *increased* arousal and responsiveness to emotional stimuli and threats, as seen in anxiety and stress-related disorders.

## Supplementary information

Supplementary Material

## Data Availability

Processed pupil (including time series data), ECG and self-report data are openly available on the ETH Library Research Collection under 10.3929/ethz-c-000795862.
