# Supplementary material for: Pupil-based arousal self-regulation: impact on physiological and affective responses to emotional stimuli
Source: Transl Psychiatry. 2026 Mar 19;16:191. doi: 10.1038/s41398-026-03937-3 (PMC13039922; doi:10.1038/s41398-026-03937-3)
Supplement: Supplementary file 1 — Supplementary Material [file 41398_2026_3937_MOESM1_ESM.docx]

Supplementary Material

1. Supplementary Methods
   1. **Pupil-based biofeedback (session 1-3)**

Participants were trained with a pupil-based biofeedback (pupil-BF) approach containing three training sessions conducted on three separate days as described in Meissner et. al^1^. In brief, they sat in a shielded room on a chair with their chin placed in a chinrest to ensure a stable head position. Participants’ eyes were ~65cm away from the eye tracker (Tobii TX300, Tobii Technology AG, Stockholm, Sweden) that was positioned below the screen (240B7QPJ, resolution: 1680x1050; Philips, Amsterdam, Netherlands) to allow for optimal eye tracking and measurement of pupil size. Participants were instructed to look at the fixation dot displayed at the center of the screen. Pupil diameter and eye gaze data of both eyes were sampled at 60 Hz using the Tobii TX300 SDK for MATLAB version 3 and MATLAB 2013a. At the start of each session, the eye tracker was calibrated using a 5-point calibration. Throughout the experiment, we ensured that all used colors were isoluminant to the grey background using a previously described approach^1^ (https://www. w3.org/Graphics/Color/sRGB). Stimulus presentation throughout the experiment was controlled using the MATLAB-based presentation software Psychtoolbox 3.0.17.

Participants received online and post-trial visual feedback on their pupil modulation performance in training session 1 and 2. In each training session, three up- and three downregulation blocks were performed, each consisting of ten trials (30 UP/30 DOWN trials per session). Each trial started with the written instruction “INCREASE” (i.e., for UP) or “DECREASE” (i.e., for DOWN) displayed in green for 2s on a grey background followed by a baseline phase of 7s. During the baseline phase, participants were instructed to silently count backwards in steps of four to maintain a controlled mental state while seeing a green fixation dot in the center of a screen surrounded by a dashed green circle. Then, a 15s modulation phase followed, requiring participants to use mental strategies to up- or downregulate their own pupil size while receiving online pupil-BF indicated by a moving green circle centered around the fixation dot. This moving circle showed pupil size relative to a dashed circle representing the mean pupil size of the baseline phase. We accounted online for artifacts caused by eye blinks, physiological and measurement-based noise as previously described^1^. This modulation phase was followed by post-trial performance feedback for 2s. Here, valid pupil diameter samples were averaged across the modulation phase, the maximum change was extracted and displayed on the screen in black. The display of the average was color-coded: If participants successfully modulated pupil size into the required direction, the circle indicating the average change was shown in green. If pupil size modulation was not successful, the circle was depicted in magenta. After a break of 5s, a new trial started. Following each block, participants could take a short self-determined break before they continued with the next block. In session 3, the training was adapted as follows: (1) online feedback was removed, and participants only received post-trial performance feedback. Here, the baseline phase was indicated by an ‘=’ sign above the fixation dot on the screen, changing to an ‘x’ as soon as the modulation phase started with task phases being presented in magenta instead of green; (2) the modulation phase was prolonged to 30s whereas all other phases were kept at the same length. For consistency between training sessions, we only report the first 15s of self-regulation. This prolongation was implemented since we included blood pressure measurements requiring longer recording durations as well as electroencephalography (EEG) recordings. Blood pressure data are not reported here. For EEG data, see^2^. All participants underwent pupil-BF training sessions within a period of seven days. The training sessions took place roughly at the same time of the day to keep circadian influences constant. Prior to training day 1, we determined the dominant eye of each participant using the Miles test^3^ since the displayed feedback during training was determined by the data recorded of the dominant eye. After the pupil-BF training sessions, we conducted a debriefing in which participants reported in their own words which mental strategies they used for up- and downregulation, respectively.

- 1. **Emotion-inducing task: Stimuli and block design**

During the emotion-inducing session, Pupil-BF or a non-regulatory control condition was combined with the presentation of negative or neutral sounds. Therefore, 120 sounds, 60 neutral and 60 negative, derived from the IADS-2^4^ were presented to the participants via headphones (Panasonic Marketing Europe GmbH, Wiesbaden, Germany). Each of the three conditions (UP, DOWN and NON-REG) was presented in eight blocks of five trials, resulting in 24 task blocks and a total of 120 trials. The blocks were presented in a pseudo-randomized order (every condition was presented once before it could be presented again). To avoid habituation, negative and neutral sounds were distributed into three sets of 20 sounds each, one set for every condition. These sets were matched for maximum volume (set 1 (mean ± SD): 70 ± 6.8 dB, set 2: 70 ± 7.5 dB, set 3: 70 ± 6.8 dB), mean arousal (negative: set 1: 6.7 ± .8, set 2: 6.6 ± 1.1, set 3: mean = 6.7 ± .6; neutral: set 1: 5.0 ± 1.0, set 2: 4.9 ± .7, set 3: 5.0 ± .8) and mean valence of the database (negative: set 1: 2.8 ± .8, set 2: 2.8 ± .7, set 3: 2.8 ± .7; neutral: set 1: 5.2 ± .6, set 2: 5.3 ± .6, set 3: 5.3 ± .7). The assignment of each set to the three self-regulation conditions was counterbalanced across participants. The sequence of negative and neutral sounds was pseudo-randomized within each condition with the restriction that no more than three sounds of the same category (i.e., neutral or negative) were played after each other. This order was kept constant across all conditions.

- 1. **Pupil size and cardiovascular measurements**

Pupil data was collected in the same manner as described in 1.1 Pupil-based biofeedback (session 1-3). Electrocardiography (ECG) and respiratory data was recorded using the Biopac MP 160 system and the accompanying AcqKnowledge software (Biopac Systems Inc., USA). ECG was recorded continuously and sampled at 1000Hz from two electrodes, one attached to the left lower rib and one under the right clavicle. An additional electrode attached to the left clavicle was used as a reference. Respiration was measured continuously using a respiratory belt (Biopac Systems Inc., USA).

- 1. **Offline processing of pupil data**

(Pre-)processing of pupil data of all sessions was conducted using MATLAB R2020b (MathWorks, Inc., Natick, MA, USA). Recorded pupil size and gaze data of each trial were visually inspected to ensure that participants properly engaged on the fixation cross throughout the experiment and that they did not use eye movement related strategies (e.g., squinting or vergence movements). Trials were excluded from further analysis, if violations such as large eye movements/saccades (i.e., deviations > ~16° and ~10° of visual angle on the x- and y-axes, respectively) potentially affecting the validity of the pupil data^5^ were identified. Then, pupil data of both eyes were systematically preprocessed following the guidelines and standardized open-source pipeline published by Kret and Sjak-Shie^6^. Invalid pupil diameter samples such as dilation speed outliers and large deviation from trend line pupil size were removed using a median absolute deviation (MAD; with the multiplier set to 12^6^). Further, temporally isolated samples with a maximum width of 50ms that border a gap larger than 40ms were removed. Next, mean pupil size time series were generated from both eyes which were used for all further analyses reported. The data were resampled with interpolation to 1000Hz and smoothed using a zero-phase low-pass filter with a recommended cutoff frequency of four ^6^. The resulting data were inspected and trials with more than 50% of missing data points across baseline and modulation phases were excluded (training session 1: upregulation = 3%; downregulation = 8%; z = 2.12; *p* = 0.03; r = 0.44; training session 3: upregulation = 3%; downregulation = 6%; z = 1.33; *p* = 0.14; r = 0.28; emotion induction session: upregulation = 3%; downregulation = 3%; control = 3%; χ^2^ = 1.02; *p =* 0.60). Finally, preprocessed pupil diameter was corrected relative to baseline by using a previously recommended subtractive baseline approach^7^. Here, we computed the mean pupil size of the last 1000ms before the start of the modulation phase of each trial and subtracted this value from each data point of the modulation phase.

- 1. **Pupil time series analysis**

*Pupil dilation responses*

To statistically compare pupil dilation responses, the data was analysed using the MATLAB-based SPM1D toolbox for one dimensional data (SPM1D version M.0.4.11; <https://spm1d.org/>), which allows statistical inference across the entire time series while accounting for temporal autocorrelation and multiple comparisons (previously applied to 1D kinematic, biomechanical or force trajectories^8,9^ as well as pupil data^1^). Because SPM1D cannot handle missing values, missing values were spline-interpolated (MATLAB function interp1). Cubic spline interpolation was chosen as it better preserves the natural variations in pupil size than linear interpolation^10^.

*Pupil dilation velocity*

Because extraction of pupil dilation velocity requires a continuous signal, trial-level pupil size time series were cubic-spline-interpolated (MATLAB interp1 function). The first derivative was determined and then low-pass-filtered using a third-order Butterworth filter, (cut-off frequency = 18Hz) to attenuate high frequency noise following Ten Brink and colleagues^11^.

- 1. **Cardiac data preprocessing**

ECG R peaks throughout the emotion-inducing session were detected automatically and, if necessary, manually corrected using the MATLAB-based toolbox Physiozoo^12^. ECG segments consisting of poor quality including non-detectable peaks were excluded from further analyses. Resulting R-R intervals for which both R peaks were occurring in the baseline, modulation or sound phase of the pupil-BF training were extracted and further processed in MATLAB (R2022b). Unfortunately, data of one participant was of bad quality for more than 50% of the data segments, leading to an exclusion and n = 22 data sets for the final data analyses. Heart rate, reflecting cardiovascular dynamics controlled by an interplay between the sympathetic and parasympathetic nervous system, was calculated by dividing 60 through the respective R-R intervals (divided by 1000) of the baseline, modulation, and sound phases.

- 1. **Control analysis of average baseline values for heart rate and pupil size**

To test whether baseline values for pupil size and heart rate already differed between the self-regulation conditions (UP, DOWN and NON-REG) during baseline phases (i.e., pre-modulation), we averaged pupil size over the last second of the baseline measurement or heart rate over the entire baseline phase, respectively, for each self-regulation and sound condition (negative and neutral). Depending on whether the data was normally distributed (Shapiro Wilk test; p > .05), the data was either subjected to a repeated-measures ANOVA with the within-subject factor self-regulation (UP, DOWN, NON-REG) or to a Friedman ANOVA. Sphericity was assessed using Mauchly’s sphericity test and violations were accounted for with the Greenhouse-Geisser correction. In case of significant effects, we used sequential Bonferroni correction for post-hoc comparisons^13^ (for results, see Supplementary Fig. 2).

- 1. **Emotion regulation strategy use, anxiety levels and pupil self-regulation success**

To test whether emotion regulation strategy use (assessed via the ERQ) relates to pupil-BF training success, we computed Spearman’s rho correlation coefficients between the improvement in pupil modulation indices (MI; i.e., UP – DOWN) from session 1 to session 3 (i.e., MI_session3_-MI_session1_) and the scores for the emotion regulation strategies of expressive suppression and cognitive reappraisal, respectively. Similarly, we computed Spearman’s rho correlation coefficients between the improvement in the MI and trait and state anxiety scores. However, neither self-reported habitual emotion regulation strategy use (cognitive reappraisal; *p* = 0.59; expressive suppression scores; only trend-level; rho = -0.39; *p* = .07; uncorrected) nor anxiety levels (all p > .26) were related to self-regulation training success.

- 1. **Habitual emotion regulation strategy use, anxiety, and pupil dilation responses towards sounds**

Finally, we investigated whether individuals’ general habitual emotion regulation use or trait anxiety levels were predictive of pupil dilation responses to sounds. We assumed that (i) emotion regulation strategy use may be related to differences in physiological responses between self-regulation conditions (i.e., emotion regulation; UP – NON-REG and NON-REG – DOWN) whereas (ii) anxiety may be rather related to differences in physiological responses between sound categories (i.e., emotional reactivity; negative-neutral). First, we computed separate regression models where pupil dilation differences (i.e., UP – NON-REG and NON-REG – DOWN for negative and neutral sounds, respectively) were entered as dependent variables and emotion regulation strategy use (i.e., expression suppression and cognitive reappraisal scores) were entered as predictors, while controlling for participants’ sex. Second, separate regression models were setup where pupil dilation differences (i.e., negative-neutral) were entered as dependent variable and trait anxiety scores were entered as predictors, while controlling for participants’ sex. If the dependent variable deviated from normal distribution (Shapiro-Wilk test p < .05), we conducted non-parametric correlation analyses (i.e., Spearman’s Rho correlation coefficients). For each hypothesis, significant p-values were corrected for multiple comparisons using sequential Bonferroni correction^13^.

- 1. **Spearman’s attenuation correction**

Originally, Spearman’s attenuation correction results in an adjusted correlation index of R^2^_adj_, which considers the actual R^2^ of the analysis divided by the reliability of variable x (in our case the reliability of pupil self-regulation) multiplied by the reliability of variable y (i.e., intensity of affect self-ratings):

$\left( 1 \right) \frac{R^{2}}{\mathrm{reliability}\left( x \right)\times reliability(y)}$ = $R_{\mathrm{adj}}^{2}$

This attenuation correction, however, assumes a reliability of both measures of 1. The modified Spearman’s attenuation correction results in a corrected estimation of R^2^ (R^2^_corr_) by allowing for an adjustment of this perfect reliability by setting a maximum assumed reliability of variable x and y respectively.

$${\left( 2 \right) R}_{\mathrm{corr}}^{2}= R_{\mathrm{adj}}^{2}\times reliability\left( x_{\mathrm{ass}} \right) \times reliability(y_{\mathrm{ass}})$$

In our dataset, we estimated the reliability in our single-session affect ratings by using a split-half approach, where we randomly split the trial-based affect ratings for negative sounds of each individual participant into half for each self-regulation condition, averaged the ratings of each of the datasets for each participant and calculated a Pearson’s correlation coefficient for each self-regulation condition between the two halves. This resulted in a correlation coefficient of r = 0.67 for upregulation trials, r = 0.79 for downregulation trials, and r = 0.81 for control trials. Averaging across conditions, this would result in r = 0.76. In general, self-regulation performance once trained is usually relatively stable across different time points. In an independent sample of n = 26 (unpublished data) where we specifically focused on downregulation, downregulation performance (i.e., a decrease in pupil size as compared to a baseline phase) in one session explained 47% of the downregulation in a follow-up session (R^2^ = 0.47; p = 0.001)). Here, we set the assumed reliability for both variables to a maximum of 0.9.

1. Supplementary Figures


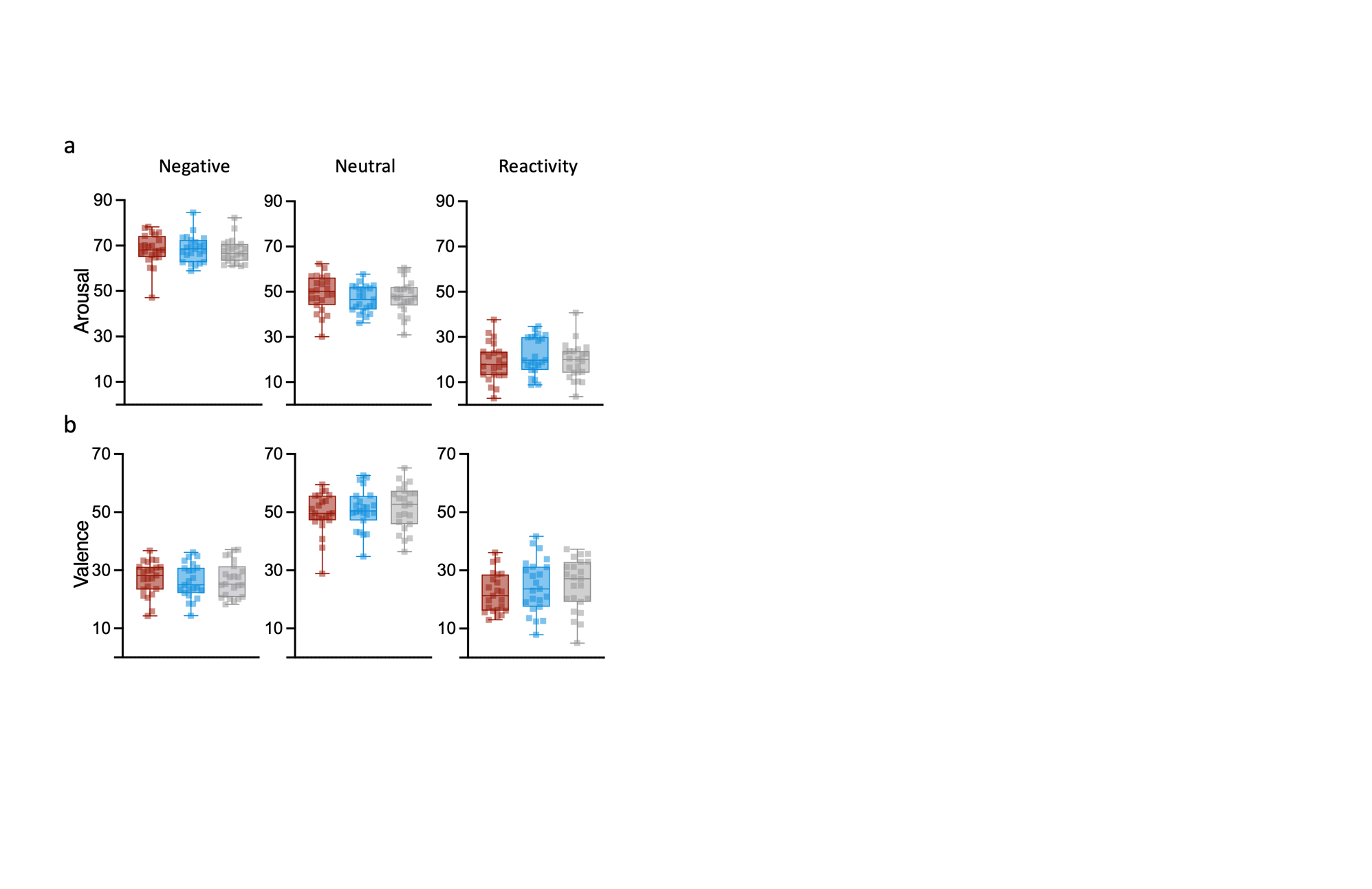


**Supplementary Figure 1.** Behavioral self-ratings of arousal and valence. Arousal (**a**), and valence (i.e., pleasantness) (**b**) rated on a visual analogue scale following negative (left panels) and neutral sound presentation (middle panels) during upregulation (red), downregulation (blue), and non-regulatory control trials (grey). As hypothesized, negative sounds were rated as more arousing and less pleasant than neutral sounds across all self-regulation and control conditions (arousal UP: z = 4.20; p < .001; r = 0.88; DOWN: z.= 4.20; p < .001; r = 0.88; NON-REG: z = 4.20; p < .001; r = 0.88; valence UP: z = -4.20; p < .001; r = 0.88; DOWN: z = -4.20; p < .001; r = 0.88; NON-REG: z = -4.20; p < .001; r = 0.88). However, there were no significant effects of self-regulation on arousal or valence ratings for neither negative nor neutral sounds (negative: arousal: p = .30; valence: p = .57; neutral: arousal: p = .12; valence: p = .40), nor a significant effect on differences between self-regulation conditions (i.e., to test for an interaction effect between self-regulation condition and sound category; right panels) in valence ratings between negative and neutral sounds (p = 0.74). There was a trend for an effect of self-regulation on the difference in arousal between negative and neutral sounds (i.e., emotional reactivity; χ2 = 5.30; p = .07), suggesting that pupil-based self-regulation (and especially downregulation) may have influenced arousal, primarily via the neutral sound domain. Boxplots indicate median (centre line), 25th and 75th percentiles (box), and maximum and minimum values (whiskers). Squares indicate individual participants. All post-hoc tests were two-tailed and corrected for multiple comparisons.


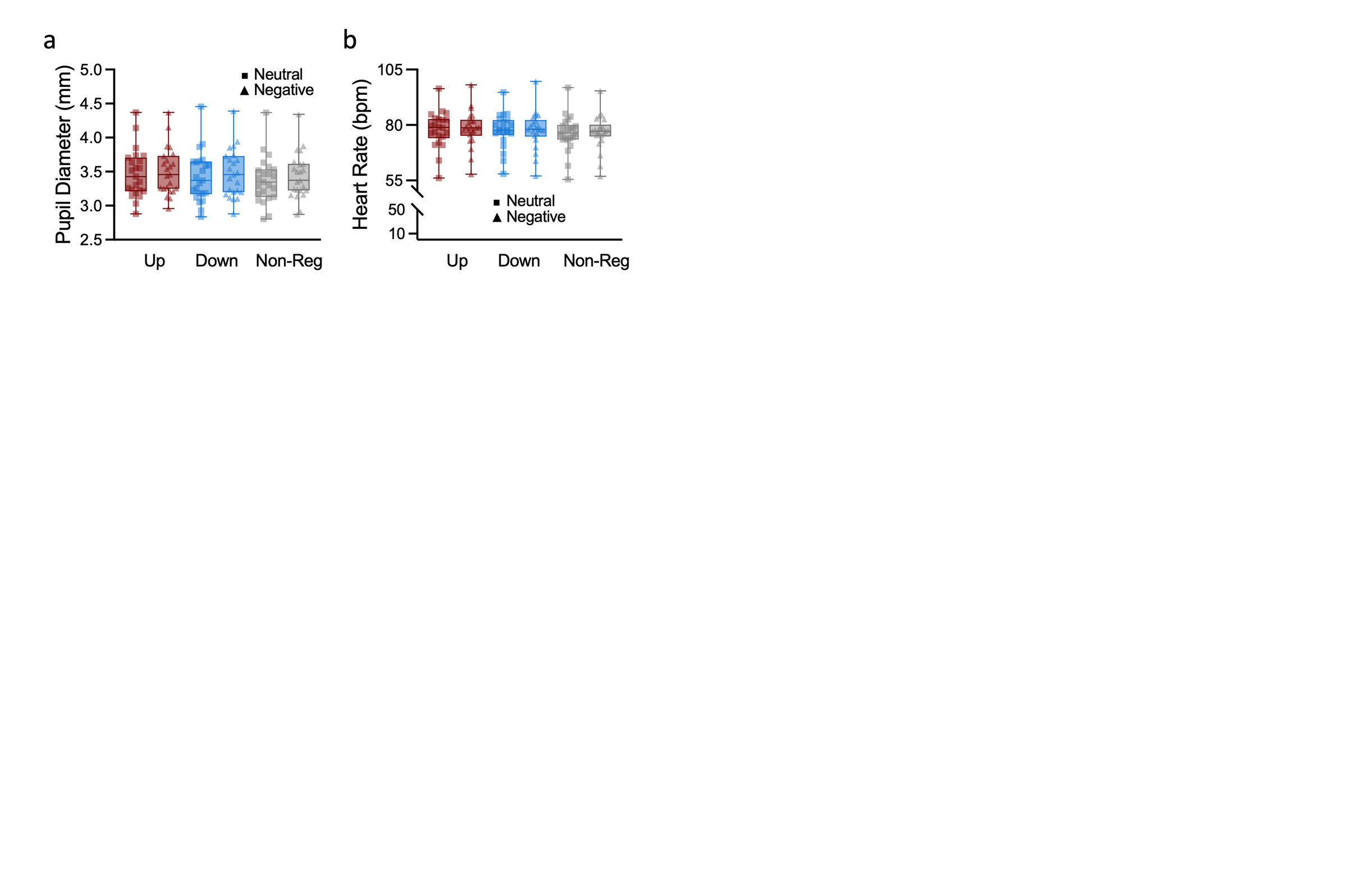


**Supplementary Figure 2: Average baseline pupil size and heart rate.** (a) Pupil size averaged for upregulation (red), downregulation (blue) and non-regulatory control trials (grey) across all participants (n = 23) for the baseline phases prior to self-regulation and sound presentation. Repeated measures ANOVA revealed a significant effect of self-regulation condition F(2,44) = 11.78; p < 0.001; η_p_^2^ = 0.35; 95%-CIη_p_^2^ [0.12;0.51]) and sound (F(1,22) = 39.69; p < 0.001; η_p_^2^ = 0.64; 95%-CIη_p_^2^ [0.35;0.77]). These effects were largely driven by bigger pupil size during upregulation compared to the non-regulatory control (p < .001) and downregulation trials (p = 0.03; down > non-regulatory control; p = 0.033) and during negative as compared to neutral sounds (p < .001). The sound*condition interaction did not reach significance (p = 0.31). These differences in baseline are most likely due to short intertrial-intervals after feedback presentation, so that pupil diameter would not fully normalize back to baseline. However, as the baseline pupil diameter was highest for the UP condition, this increased the difficulty to upregulate the already enlarged pupil diameter and renders our results reported in the main manuscript even more conservative. Heart rate was averaged across all participants (n = 22) for upregulation (red), downregulation (blue), and non-regulatory control trials (grey) for the baseline phases prior to self-regulation and sound presentation. Even though we did not anticipate differences in heart rate during the baseline period, heart rate did differ prior to sound presentation between self-regulation conditions (F(2,42) = 13.82; p < 0.001; η_p_^2^ = 0.40; 95%-CIη_p_^2^ [0.15;0.55]). Similarly to baseline pupil diameter, these effects were mainly driven by higher heart rates during upregulation and downregulation as compared to non-regulatory control trials (all p < .001). There were no other significant effects (main effect sound: p = .10; sound*self-regulation interaction: p = .89). Boxplots indicate median (centre), 25th and 75th percentiles (box), maximum and minimum values (whiskers). Squares and triangles represent individual data.

**
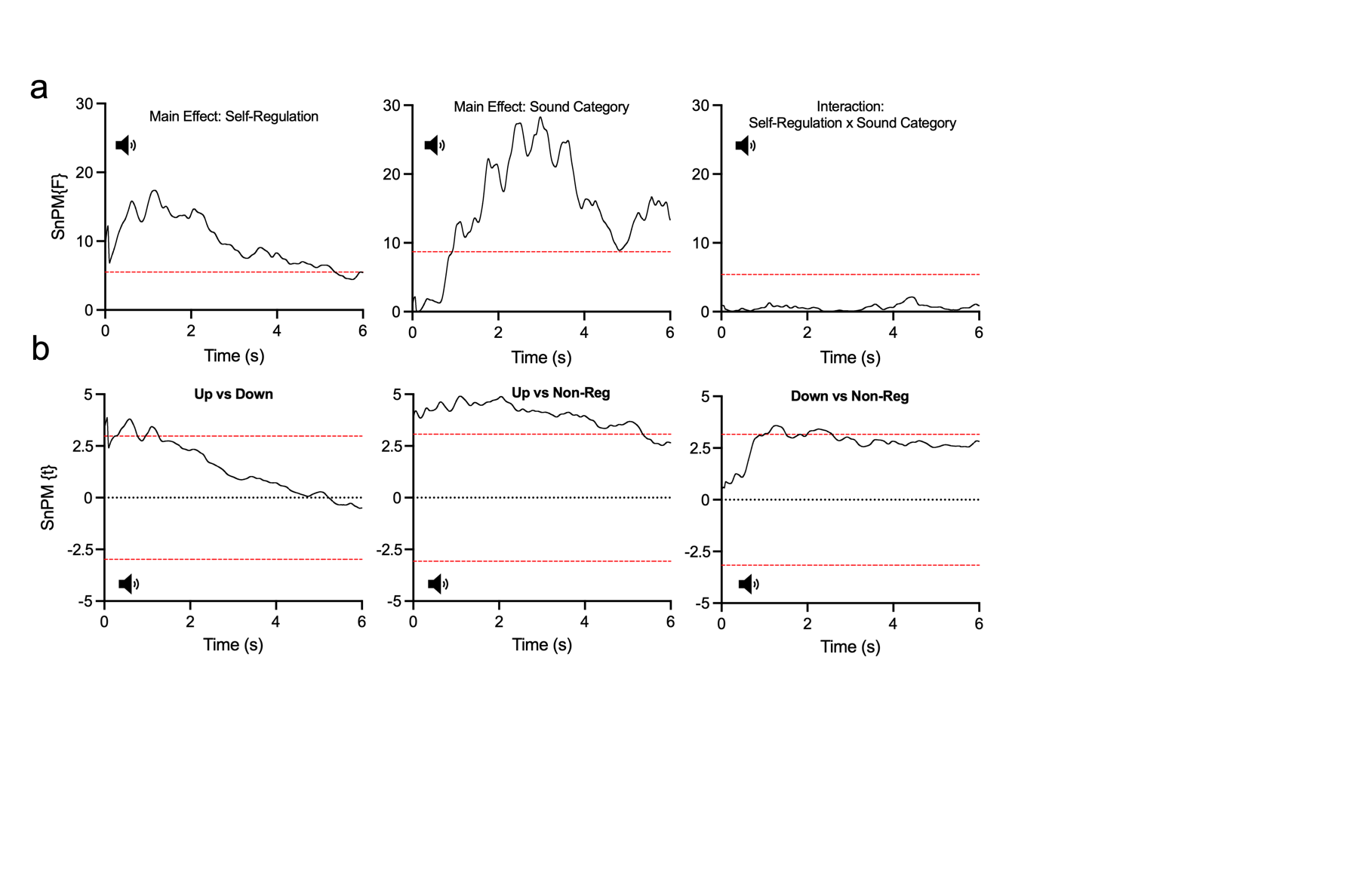
**

**Supplementary Figure 3. SPM1D output of the sound-evoked pupil dilation time series analysis.** (a) Statistical parametric maps (SPM{F}) showing the main effect of self-regulation (left panel), the main effect of sound (middle panel), and the interaction effect of self-regulation and sound condition (right panel) across the pupil dilation time course (baseline-corrected to the 200ms preceding sound onset). For the main effect of self-regulation, two significant clusters exceeded the critical threshold (F* = 5.506; p = 0.001), indicating that pupil dilation responses differed significantly depending on self-regulation condition. For the main effect of sound, one cluster was detected that exceeded the critical threshold (F* = 8.712; p = 0.001), indicating that pupil responses were higher to negative compared to neutral sounds. (b) SPM{t} maps of post-hoc contrasts for the main effect of self-regulation, collapsed across sound condition. Upregulation evoked significantly larger pupil dilation than downregulation (t* = 2.98; p = 0.002; Bonferroni-corrected α = 0.0167; left panel) and non-regulatory control trials (t* = 3.07; one cluster, p = 0.001; middle panel). Downregulation evoked significantly larger pupil dilation than non-regulatory control trials (t* = 3.16; p = 0.001; right panel). Red dashed lines indicate the critical threshold for significance (Clusters exceeding this threshold are considered significant).


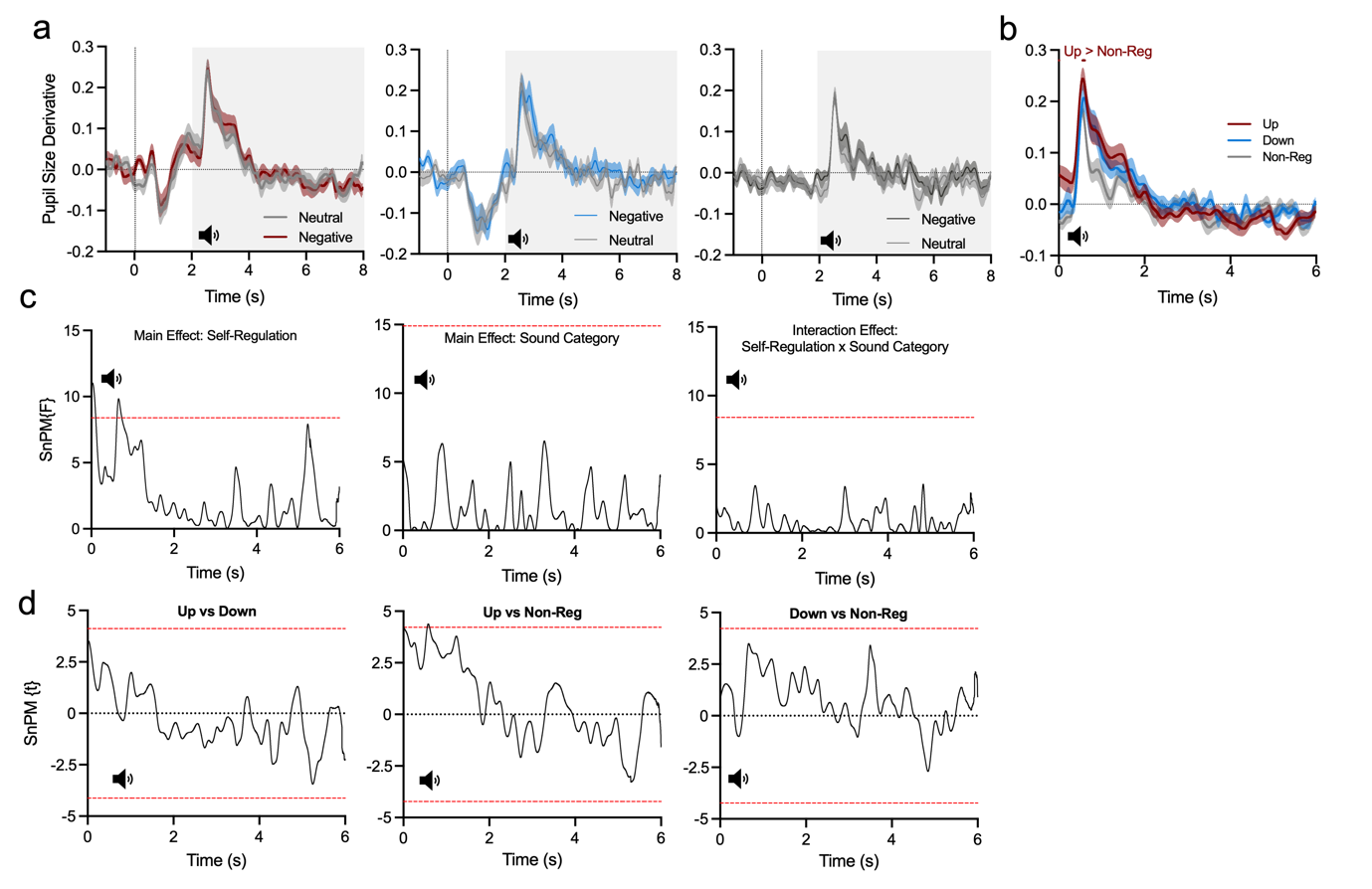


**Supplementary Figure 4. Pupil dilation velocity over time.** (a) Average changes in pupil dilation velocity over time to negative and neutral sound trials during upregulation (left panel), downregulation (middle panel) and non-regulatory control (right panel). Self-regulation starts at t=0s (dashed vertical line), sound presentation starts at t=2s (indicated by sound icon and grey box). (b) Average pupil dilation velocity over time shown for the sound presentation phase for the different self-regulation conditions (upregulation in red, downregulation in blue, and non-regulatory control trials in grey), collapsed across sound conditions (i.e., neutral and negative). (c) Statistical parametric maps (SPM{F}) showing the main effect of self-regulation (left panel), the main effect of sound (middle panel), and the interaction effect of self-regulation and sound condition (right panel) during sound presentation over time. Statistical analysis of the main effect of self-regulation revealed two significant clusters where pupil dilation velocity differed significantly between self-regulation conditions (F* = 8.39; p = 0.001). No significant clusters were detected for the main effect of sound and the interaction of sound and self-regulation (p >0.05). (d) SPM{t} maps of post-hoc contrasts for the main effect of self-regulation, collapsed across sound conditions. Pupil dilation changes were faster for upregulation than non-regulatory control trials for two clusters (t* = 4.22; two clusters with p = 0.008 and p = 0.006; Bonferroni-corrected α = 0.0167; left panel). No significant differences in pupil dilation velocity were found between upregulation and downregulation (middle panel) and downregulation and non-regulatory control (right panel; all p > 0.0167). In (a) and (b) shaded areas indicate s.e.m. and the red horizontal lines denote time clusters with significant differences between self-regulation conditions. In (c) and (d), red dashed lines indicate the critical threshold for significance (i.e., clusters exceeding this threshold are considered significant).

**
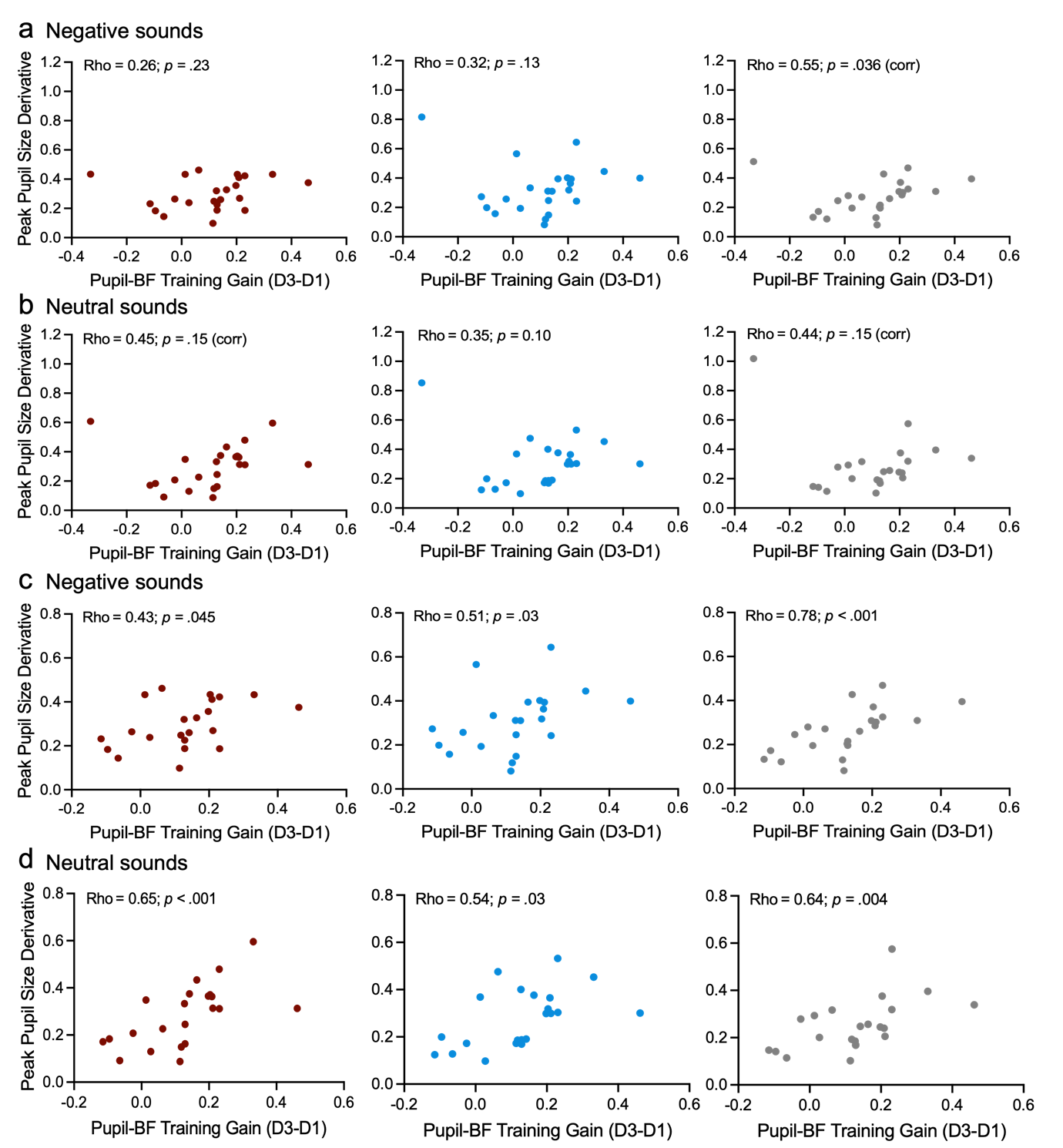
Supplementary Figure 5. Pupil-BF training success and pupil dilation velocity.** Peak pupil dilation velocities extracted from the first derivative shown in Supplementary Figure 4 for negative (a) and neutral sounds (b) during upregulation (left panel), downregulation (middle panel) and non-regulatory control trials (right panel) and their correlation with Pupil-BF training gain (i.e., pupil modulation index_sesssion3_-pupil modulation index_session1_). Including all participants, peak velocity to negative sounds was positively related to pupil-BF training gain for non-regulatory control trials (a, right panel; Rho = 0.55, *p* = 0.036; corrected for multiple comparisons using sequential Bonferroni correction), indicating that the larger the pupil-BF training gain from day 1 to day 3, the faster the pupil dilation to sounds. For self-regulation trials, these variables were largely unrelated (a,b, all *p* > .15, corrected). This was mainly driven by one outlier with a very low training success which was removed in (c) and (d). Significant positive relationship between pupil-BF training gain and peak pupil velocity for negative (c) and neutral (d) sounds during upregulation (left panel), downregulation (middle panel) and non-regulatory control trials (right panel; all corrected for multiple comparisons using sequential Bonferroni correction), indicating that the higher the pupil-BF training gain, the faster peak pupil dilation responses to sounds, irrespective of self-regulation and sound conditions.


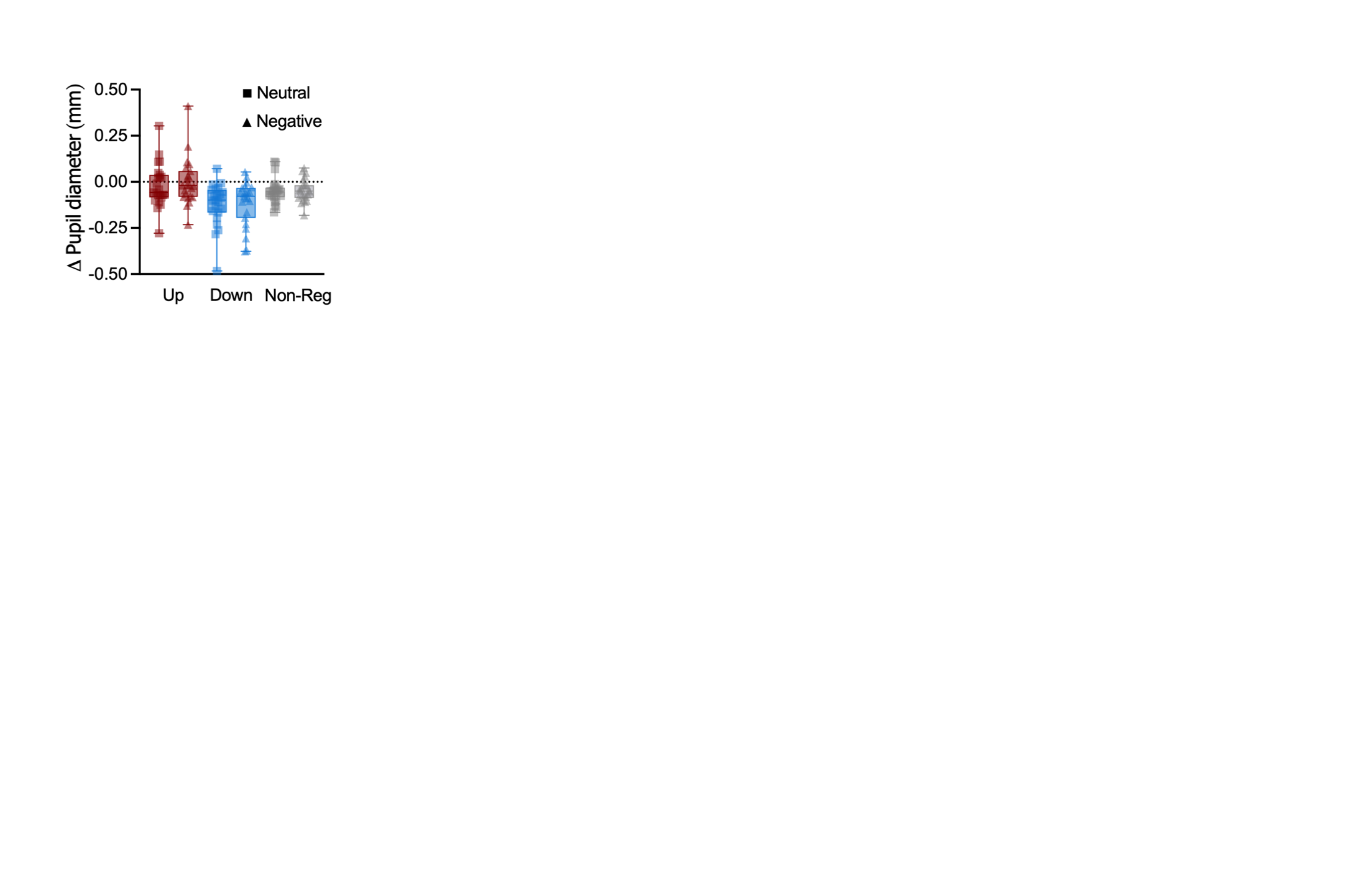


Supplementary Figure 6. Pupil self-regulation prior to sound onset. Baseline-corrected pupil size averaged across 200ms before sound onset during upregulation (red), downregulation (blue) and non-regulatory control (grey). Analyses revealed a significant main effect of self-regulation condition (Friedman ANOVA: χ2 = 12.09; p = .002). This difference was mainly driven by a stronger decrease during downregulation as compared to upregulation (z = -3.13; p = .006; r = -0.65) and non-regulatory control trials (z = -2.89; p = .008; r = -0.60). Upregulation trials, on the other hand, were not significantly different to non-regulatory control trials 200ms before tone onset (z = 1.64; p = .10). Boxplots indicate median (centre), 25^th^ and 75^th^ percentiles (box), maximum and minimum values (whiskers). Squares and triangles represent individual data.


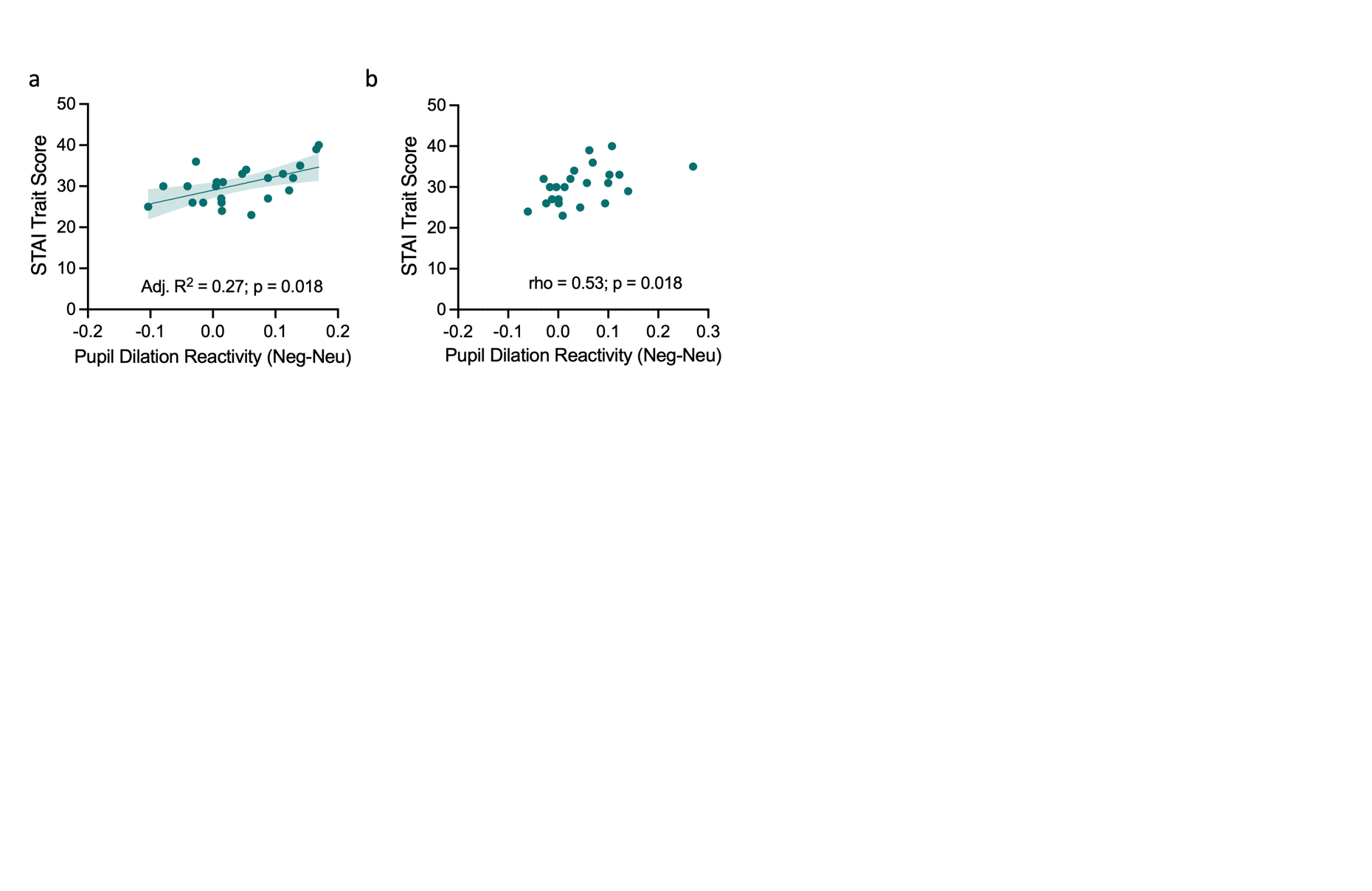


Supplementary Figure 7. Higher trait anxiety is associated with larger physiological responses to sounds. (A) Higher trait anxiety levels predicted larger maximal pupil dilation responses to negative as compared to neutral sounds (i.e., emotional reactivity; negative-neutral) during upregulation (R = 0.55; adjusted R2 = 0.27; p = 0.018; but not during downregulation; p >.86). (B) Similarly, higher trait anxiety was significantly associated with larger pupil dilation responses to negative as compared to neutral sounds during non-regulatory control trials (rho = 0.53; p = 0.018). There was no significant link between emotion regulation strategy use (i.e., cognitive reappraisal and expressive suppression) and pupil dilation (only trend-level effect uncorrected for multiple comparisons, R^2^ = 0.38; adjusted R^2^ = 0.14; p = 0.08, for expressive suppression as a predictor for the difference between pupil dilation responses during downregulation and non-regulatory control). Shaded areas indicate the 95%-CI.

**References**

1 Meissner SN, Bächinger M, Kikkert S, Imhof J, Missura S, Carro Dominguez M *et al.* Self-regulating arousal via pupil-based biofeedback. *Nat Hum Behav* 2024; **8**: 43–62.

2 Weijs ML, Missura S, Potok-Szybińska W, Bächinger M, Badii B, Carro-Domínguez M *et al.* Modulating cortical excitability and cortical arousal by pupil self-regulation. *Nat Commun* 2025; **16**: 4552.

3 Miles WR. Ocular dominance in human adults. *Journal of general psychology* 1930; **3**: 412–430.

4 Bradley MM, Lang PJ. The International Affective Digitized Sounds (2nd Edition; IADS-2): Affective ratings of sounds and instruction manual. Technical report B-3. *University of Florida, Gainesville, Fl* 2007.

5 Hayes TR, Petrov AA. Mapping and correcting the influence of gaze position on pupil size measurements. *Behav Res Methods* 2016; **48**: 510–527.

6 Kret ME, Sjak-Shie EE. Preprocessing pupil size data: Guidelines and code. *Behav Res Methods* 2019; **51**: 1336–1342.

7 Mathôt S, Fabius J, Heusden E Van, Stigchel S Van Der. Safe and sensible preprocessing and baseline correction of pupil-size data. *Behav Res Methods* 2018; **50**: 94–106.

8 Pataky TC, Robinson MA, Vanrenterghem J. Vector field statistical analysis of kinematic and force trajectories. *J Biomech* 2013; **46**: 2394–2401.

9 Pataky TC, Vanrenterghem J, Robinson MA. Zero- vs. one-dimensional, parametric vs. non-parametric, and confidence interval vs. hypothesis testing procedures in one-dimensional biomechanical trajectory analysis. *J Biomech* 2015; **48**: 1277–1285.

10 Fink L, Simola J, Tavano A, Lange E, Wallot S, Laeng B. From pre-processing to advanced dynamic modeling of pupil data. *Behav Res Methods* 2024; **56**: 1376–1412.

11 Ten Brink AF, Heiner I, Dijkerman HC, Strauch C. Pupil dilation reveals the intensity of touch. *Psychophysiology* 2024; **61**. doi:10.1111/psyp.14538.

12 Behar JA, Rosenberg AA, Weiser-Bitoun I, Shemla O, Alexandrovich A, Konyukhov E *et al.* PhysioZoo: A novel open access platform for heart rate variability analysis of mammalian electrocardiographic data. *Front Physiol* 2018; **9**: 1–14.

13 Holm S. A Simple Sequentially Rejective Multiple Test Procedure. *Scandinavian Journal of Statistics* 1979; **6**: 65–70.
